# Cross‐linked Triblock Peptide Capsules as Potential Oxygen Carriers

**DOI:** 10.1002/open.202300282

**Published:** 2024-03-12

**Authors:** Huayang Feng, Jürgen Linders, Miriam Cantore, Jonas Fabrizi, Annika Kirsten, Sascha Myszkowska, Eva Hillen, Florian Uteschil, Sebastian Buchholz, Andrea Hermsen, Maria Davila Garvin, Katja Bettina Ferenz, Christian Mayer

**Affiliations:** ^1^ Institute for Physical Chemistry CeNIDE University of Duisburg-Essen 45141 Essen Germany; ^2^ Institute of Physiology University Hospital Essen CeNIDE University of Duisburg-Essen 45147 Essen Germany; ^3^ Institute of Inorganic Chemistry and Structural Chemistry Heinrich-Heine-Universität Düsseldorf 40225 Düsseldorf Germany; ^4^ Applied Analytical Chemistry University of Duisburg-Essen 45141 Essen Germany; ^5^ Institute for Technical Chemistry University of Duisburg-Essen 45141 Essen Germany; ^6^ Department of Chemistry and ILOC Niederrhein University of Applied Sciences 47805 Krefeld Germany

## Abstract

Perfluorodecalin (PFD)‐filled capsules have been studied for over 15 years as artificial oxygen carriers. However, none of these capsules combines good biocompatibility, good mechanical stability and dispersion stability. Here we propose to use synthetic triblock peptides containing a central block of cysteine units as a cross‐linking shell material for capsules with both good biocompatibility and stability. Together with outer aspartate units and inner phenylalanine units, the resulting amphiphilic triblock peptides can encapsulate PFD efficiently to prepare capsules with a suitable diameter, a certain mechanical strength, a large diffusion constant, fast gas exchange rates, and little cytotoxicity. Given the above advantages, these PFD‐filled peptide capsules are very promising as potential artificial oxygen carriers.

## Graphical abstract

We report the synthesis of perfluorodecalin (PFD)‐filled triblock peptide capsules. The capsules exhibit a suitable diameter, a certain mechanical strength, a large diffusion constant, fast gas exchange rates, and little cytotoxicity. Given the above advantages, these PFD‐filled peptide capsules are very promising as potential artificial oxygen carriers.

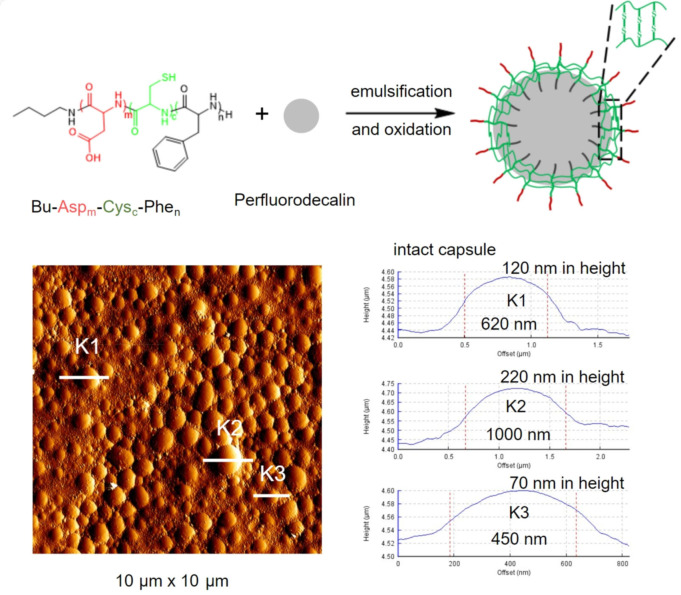

## Introduction

Thousands of patients receive red blood cell concentrates every day to maintain essential functions such as oxygen transport.^[1]^ However, there may be severe blood shortage in the future due to demographic changes or due to infection risks. Therefore, artificial oxygen carriers are being developed in order to reduce the dependency on red blood cell concentrates.^[1]^ Perfluorodecalin (PFD) is extensively studied as a functional content of artificial oxygen carriers because it exhibits relative biological inertness, good oxygen dissolving capacities and short organ retention times. However, perfluorodecalin needs to be encapsulated or emulsified before intravenous application because it is not miscible with blood medium.^[2]^

Therefore, various polymeric materials were studied by our group to encapsulate PFD in order to prepare capsules and we assessed their physio‐chemical properties and biocompatibilities. Initially, we investigated synthetic polymers as shell materials to encapsulate PFD, including poly(lactic‐co‐glycolic acid) (PLGA) and poly(n‐butyl‐cyanoacrylate) (PBCA). PLGA capsule walls not only show a mechanical stability comparable to that of red blood cells, but also have sufficient permeability to allow oxygen exchange in an aqueous environment.^[3]^ By coating with poly(ethylene glycol) PEG and stabilizing with 1 % poly(vinyl alcohol) PVA, PLGA capsules exhibit a circulatory half‐life time of about 1 h.^[4]^ However, severe side effects such as organ damage and hypotension occurred after intravenous infusion of large amounts of PFD‐filled PLGA capsules.^[5]^ PBCA capsules (diameters of 400–800 nm) were much smaller than PLGA capsules (diameters of 1.5 μm), and their dispersions showed good oxygen carrying capacities (10 % oxygen solubility at 1 bar for a formulation containing 24 vol % PBCA capsules), which is about half of the capacity of human blood at the same conditions.^[6]^ However, several undesired side effects were observed such as a transient decrease in systemic blood pressure, impairment in hepatic microcirculation, organ damage and elevation of plasma enzyme activities.^[7]^

In order to achieve a better biocompatibility of PFD‐filled capsules, biocompatible albumin was studied as shell material. Albumin nanocapsules not only showed a remarkable maximum oxygen capacity, but also were proved to avoid severe side‐effects after *in vivo* administration.^[8]^ These albumin nanocapsules were also capable of protecting a Langendorff heart (rat) during massive ischemia.^[9]^ However, a potential weakness of these albumin nanocapsules is that their capsule walls do not exhibit sufficient mechanical strength.

Synthetic peptides contain the same amino acid building blocks as albumin, but allow for adjustable properties, which is not possible with natural albumin. Therefore, using peptides as a shell material to encapsulate PFD may create capsules with both good biocompatibility and good mechanical and dispersion stability. Three years ago, our group first reported to use synthetic amphiphilic diblock peptide consisting of a hydrophilic aspartate block and a hydrophobic phenylalanine block to stabilize PFD.^[10]^ These amphiphilic diblock peptides surfactants were proven to emulsify PFD efficiently. However, the mechanical strength of these capsules was not improved, as their capsule structure is just stabilized by amphiphilic interaction at the PFD‐water interface.

**Figure 1:**
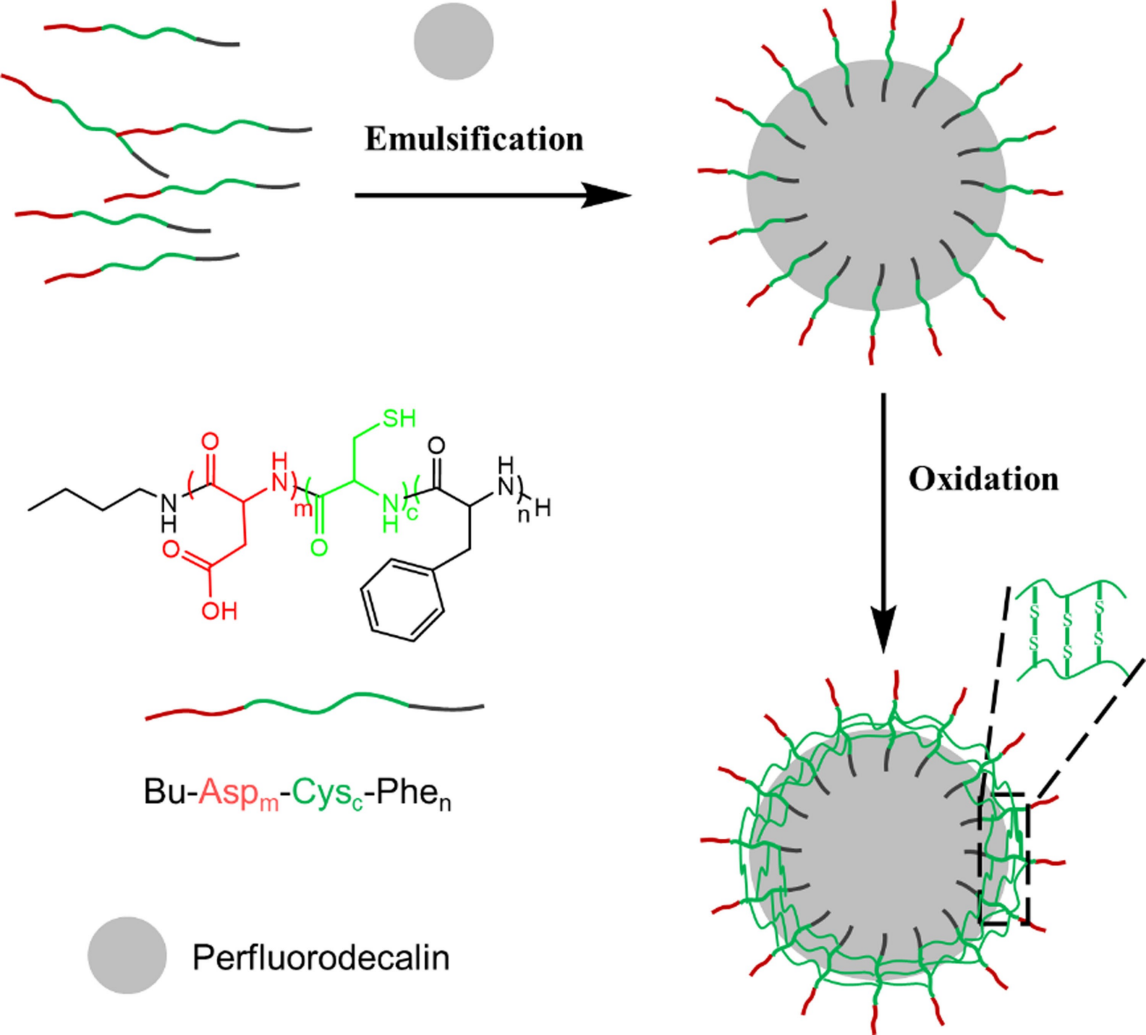
Structure of cysteine containing triblock peptide and formation of PFD‐filled peptide capsules.

In the present study, we introduce an additional central cysteine block, hereby forming a triblock surfactant structure with the capability to cross‐link via sulfur bridges. Based on this mechanism, we expect PFD‐filled capsules of high mechanical strength. The introduction of cysteine blocks can solidify the initially loosely associated capsule walls by forming networks of disulfide bonds (Figure 1). The triblock peptides are synthesized by polymerization of activated urethane derivatives (UDs) of amino acids as reported by Endo et al.^[11]^ Their chemical structures are identified using ^1^H‐NMR, MALDI‐TOF MS, and gel permeation chromatography (GPC). The morphology and size distribution of the PFD‐filled capsules are investigated with Atomic Force Microscopy (AFM) and particle tracking via Video Microscopy (VM). The Brownian motion and the gas exchange properties of the capsules are studied by Pulsed Field‐Gradient Nuclear Magnetic Resonance (PFG‐NMR) and ^19^F‐NMR. The cytotoxicity of capsules is analyzed by lactate dehydrogenase (LDH) assay. These results indicate that the novel PFD‐filled triblock peptide capsules are very promising as artificial oxygen carriers. In the future, a suitable triblock peptide may be mass‐produced by biotechnological approaches.

## Experimental Section

### Materials

4‐Benzyl‐L‐aspartate, 4‐nitrophenyl chloroformate, dithiothreitol, trifluoromethanesulfonic acid, and trifluoroacetic acid were obtained from TCI (Eschborn, Germany). L‐phenylalanine was purchased from Carl Roth (Karlsruhe, Germany). S‐carbobenzoxy‐L‐cysteine was obtained from BLD Pharmatech GmbH. Anhydrous dimethylacetamide (DMAc) was obtained from Acros (Geel, Belgium). S‐Benzyl‐L‐cysteine, hydrogen peroxide, and n‐butylamine were purchased from Alfa Aesar (Kandel, Germany). PFD was obtained from Fluorochem Chemicals (Derbyshire, UK). Methanol, ethyl acetate and diethyl ether were purchased from Fisher Scientific (Schwerte, Germany). Finally, n‐hexane and dichloromethane were obtained from VWR International (Darmstadt, Germany) and acetonitrile was purchased from Carl Roth (Karlsruhe, Germany).

### Syntheses of Triblock Peptides

All monomers, including N‐(4‐nitrophenoxycarbonyl)‐β‐benzyl‐L‐aspartate (NNPBnAsp), N‐phenoxycarbonyl‐β‐benzyl‐L‐aspartate (NPBnAsp), N‐phenoxycarbonyl‐S‐benzyl‐L‐cysteine (NPBnCys), N‐(4‐nitrophenoxycarbonyl)‐S‐carbobenzoxy‐L‐cysteine (NNPCbzCys), N‐phenoxycarbonyl‐L‐phenylalanine (NPPhe), were synthesized as reported by Kamei et al.^[12]^ They will not be further described.

### Synthesis of Bu‐BnAsp_m_

NPBnAsp (variable equivalents to the initiator) was dissolved in anhydrous DMAc. Then, n‐butylamine was added into the above solution under the protection of nitrogen. The reaction was allowed to proceed for 1–3 d at 60 °C. Subsequently, the reaction mixture was precipitated into a large amount of diethyl ether, filtered, washed with diethyl ether, and dried under vacuum for 24 h to get the final product as a white solid.

### Synthesis of Bu‐BnAsp_m_‐CbzCys_c_ or Bu‐BnAsp_m_‐BnCys_c_

Bu‐BnAsp_m_ was dissolved in anhydrous DMAc, and then NPBnCys or NNPCbzCys (variable equivalents to the initiator), was added into the above solution under the protection of nitrogen. The reaction was allowed to proceed for 1–3 d at 60 °C. Subsequently, the reaction mixture was precipitated into a large amount of diethyl ether, filtered, washed with diethyl ether, and dried under vacuum for 24 h to get the final product as a yellow solid.

### Synthesis of Bu‐BnAsp_m_‐CbzCys_c_‐Phe_n_ or Bu‐BnAsp_m_‐BnCys_c_‐Phe_n_

Bu‐BnAsp_m_‐CBzCys_c_ or Bu‐BnAsp_m_‐BnCys_c_ was dissolved in anhydrous DMAc, and then NPPhe (variable equivalents to the initiator), was added into the above solution under the protection of nitrogen. The reaction was allowed to proceed for 1–3 d at 60 °C. Subsequently, the reaction mixture was precipitated into a large amount of diethyl ether, filtered, washed with diethyl ether, and dried under vacuum for 24 h to get the final product as a white solid.

### Synthesis of Bu‐Asp_m_‐Cys_c_‐Phe_n_

Bu‐BnAsp_m_‐CBzCys_c_‐Phe_n_ was dispersed in 3 mL of CF_3_COOH, then triflic acid or 33 % HBr/CF_3_COOH (10 eq. of the protecting groups) was added into the above solution under the protection of nitrogen. The reaction was allowed to proceed for 6 h at 25 °C. Subsequently, the reaction mixture was precipitated into a large amount of diethyl ether and washed with diethyl ether. Finally, it was dried under vacuum to get the raw product as a brown solid.

Details of all these syntheses can be found in Table S1–S3.

### Synthesis of PFD‐filled Peptide Capsules

10–30 mg peptide was dispersed in 10 mL water and a few drops of sodium hydroxide (aqueous solution, 1 M) were added under stirring. 3 eq. of DTT was added to the above solution or dispersion and the mixture was stirred for half an hour to fully reduce all disulfide bridges into free thiol groups. Then 50–200 μL PFD were added to the above mixture and the mixture was treated with ultrasonication (240 W) for 3 min to obtain the emulsion. The obtained emulsion was treated with 5–10 eq. of 35 % hydrogen peroxide aqueous solution under stirring for 30 min for oxidation and crosslinking of cysteine residues to solidify the capsules. Finally, the capsule dispersion was purified by dialysis against distilled water over night.

### Characterization of the Synthesized Peptides

The structure of the synthesized peptides was characterized by ^1^H‐NMR spectra on a Bruker AVANCE III 400 MHz spectrometer using DMSO‐d_6_, CDCl_3_ and TFA‐d as NMR solvents. The molecular weight distribution of the synthesized peptides was determined using a GPC system (with a column from polymer standards service (PSS), pump PU 2008 Plus from Jasco, detector ETA‐2020 from WGE). The terminal group of the synthesized peptides was confirmed by MALDI‐TOF Mass spectrometry (Bruker Autoflex speed) as reported by Endo's group.^[13]^ The thiol group content of the synthesized peptides was obtained by detecting the amount of the product after reaction with Ellman's reagent using UV‐1900i UV‐Vis spectrophotometer from Shimadzu.

### Characterization of PFD‐filled Peptide Capsules

#### Morphology of Capsules

The morphology of the capsules was investigated using a standard AFM instrument (NanoWizard AFM from JPK Instruments, Berlin, Germany). All samples were prepared by drying a drop of the capsule dispersion on a glass surface. All measurements were conducted under the noncontact mode with noncontact mode cantilevers from NanoWorld (Neuchatel, Switzerland).

#### Size Distribution of Capsules

The size distribution of the capsules was examined by particle tracking using video microscopy as reported by Bauer et al.^[3]^ Basically, dispersed capsules were observed doing Brownian motion under optical dark field microscopy. The motion of every capsule was tracked by online video analysis and sets of lateral displacements (Δx and Δy) of every capsule over many time intervals (Δt) were recorded. The radii of all observed capsules were calculated based on the given temperature, the given solvent viscosity and the full set of displacements. Finally, a histogram of the capsule size distribution was developed.

#### Diffusion of Capsules

The average Brownian diffusion of PFD within the capsule dispersion was studied with PFG‐NMR as reported by Feng et al.^[10]^ The NMR experiments were run on a Bruker Avance Neo II 500 MHz spectrometer (Bruker BioSpin, Rheinstetten, Germany) at a fluorine resonance frequency of 470 Hz. A simple single‐pulse excitation was used to obtain fluorine line spectra. An external standard was used for a reliable determination of the chemical shifts. Spin‐lattice relaxation times were determined in a conventional inversion recovery experiment. ^19^F‐NMR diffusion experiments were run with a Bruker DIFBBI probe head. All measurements were performed at 298 K. For all measurements, the stimulated echo pulse sequence with two gradient pulses was used. Sixteen scans were accumulated for each setting. The time between two gradient pulses Δ was 25 ms. The gradients were adjusted to strengths G between 1 and 600 G/cm with a duration δ of 1.0 ms. All measurements (the full set of gradient strengths under the variation from 1 to 600 G/cm) were repeated two times.

#### Gas Exchange Properties of Capsules

The gas exchange properties of capsules were studied using ^19^F‐NMR as reported by Bauer et al.^[3]^ Briefly, for measurements under variation of the atmosphere, a home‐built sample holder is used which carries individual gas inlet capillaries for oxygen and nitrogen in order to enable an instantaneous switching between different atmospheres. The gas flow for oxygen and nitrogen was adjusted so that the overall flow rate amounted to 0.36–0.4 mL/min. The exchange between oxygen and nitrogen atmosphere and vice versa was induced by simultaneous opening and closing of the respective valves. A single pulse experiment was repeated every 20 s during the gas treatment using the approach described in the above section. The time dependent chemical shifts of the ^19^F‐NMR spectra were obtained after Fourier transformation of each resulting free induction decay. Finally, the degree of oxygen saturation in the fluorinated hydrocarbon was then calculated from the chemical shift using data from independent calibration measurements.

### Cytotoxicity of PFD‐filled Peptide Capsules

#### Cell Culture

The human proximal tubular epithelial cell line HK‐2 (American Type Culture Collection, Manassas, VA) was cultured in Dulbecco's Modified Eagle Medium (DMEM)/F‐12 with GlutaMAX (Thermo Fisher Scientific), supplemented with 10 % fetal bovine serum (FBS, Sigma), streptomycin (100 mg/ml, Thermo Fisher Scientific), penicillin (100 U/ml, Thermo Fisher Scientific) and epidermal growth factor (EGF, 10 ng/ml, PeproTech) at 37 °C in 5 % CO_2_.

For experiments, 5×10^4^ cells/well were transferred into 96‐well tissue culture plates. Control cells were incubated for 4 h with DMEM/F‐12 with GlutaMAX (no supplements), whereas treatment groups were incubated for 4 h with capsules at different concentrations (0.1 %, 0.25 %, 0.5 %, 1 %, 2.5 %, 5 %). For treatment groups, capsules were re‐suspended in cultural medium without supplements.

#### LDH Assay

To assess cell viability, lactate dehydrogenase (LDH) release was measured using the CytoTox 96 Non‐Radioactive Assay (Promega). The cytosolic enzyme LDH is released into the culture medium following cytotoxic damage‐mediated membrane disruption. LDH was quantified via a coupled enzymatic reaction in which LDH catalyzes the reaction of lactate to pyruvate through NAD+ reduction. The released NADH is used by diaphorase to reduce a tetrazolium salt into a red formazan product. Briefly, 50 μL of cell culture medium was transferred into a 96‐well plate and subjected to measurement according to manufacturer's instructions. Absorbance was measured at 490 nm with a spectrophotometer. The positive control corresponded to 10 μl lysis buffer provided from the CytoTox 96 Assay. All samples were tested in triplicate in four independent experiments. Cell toxicity was determined using the following equation:

$$Percent\;cytotoxicity\;=100\times \frac{Experimental\;LDH\;Release\;\left(OD490\right)}{Maximum\;LDH\;Release\;\left(OD490\right)}$$

#### Statistical Analyses

Statistical analyses were performed using GraphPad Prism software (Version 9.0, San Diego, CA, USA). Analyses were conducted using two‐tailed unpaired *t*‐test, two‐way ANOVA with Tukey's multiple comparisons test.

## Results and Discussion

### Peptide Synthesis and Characterization

Various cysteine‐containing triblock peptides consisting of an aspartate block (Asp), a cysteine block (Cys) and a phenylalanine block (Phe), Bu‐Asp_m_‐Cys_c_‐Phe_n_, were synthesized using differently protected monomers (see Supporting Information). The synthetic route for these peptides using N‐phenoxycarbonyl‐S‐benzyl‐L‐cysteine (NPBnCys) as monomer and n‐butylamine as initiator is shown in Figure S1. Three triblock peptides from NPBnCys were successfully synthesized, including Bu‐Asp_6_‐Cys_8_‐Phe_8_, Bu‐Asp_13_‐Cys_6_‐Phe_7_, and Bu‐Asp_7_‐Cys_15_‐Phe_2_. Their corresponding ^1^H NMR spectra are shown in the Supporting Information (Figure S2–S14). The connection of Phe block to the former diblock peptide Bu‐BnAspm‐BnCysc is easy, because the polymerization of NPBnCys yield oligo‐ or poly‐BnCys with free amine end according to the MALDI‐TOF MS result of Bu‐BnCys_17_ as shown in Figure S15, which is also consistent with the publication reported by Endo et al.^[14]^

The synthetic route of peptides using N‐(4‐nitrophenoxycarbonyl)‐S‐Carbobenzoxy‐L‐cysteine (NNPCbzCys) as monomer and n‐butylamine as initiator is shown in Figure S16. Monomer N‐phenoxycarbonyl‐S‐carbobenzoxy‐L‐cysteine (NPCbzCys) was unable to polymerize at all, possibly due to its inefficiency of conversion to NCA, as reported by Endo et al.^[15]^ Two triblock peptides were synthesized, including Bu‐Asp_31_‐Cys_4_‐Phe_3_, Bu‐Asp_40_‐Cys_5_‐Phe_5_. Their corresponding ^1^H NMR spectra are shown in the Supporting Information (Figure S17–S25). Peptides with longer cysteine block chain length are hardly obtained when NNPCbzCys was used as the monomer. The planned number of repeating units of CbzCys block is 30 for both triblock peptides. However, only 4 or 5 CbzCys were connected to Bu‐BnAsp_m_. The efficiency of connecting the Phe block to Bu‐BnAsp_m_‐CbzCys_c_ is also low. These observations may be due to the possible intramolecular termination reaction of the CbzCys block,^[16]^ as confirmed by MALDI TOF MS result of Bu‐CbzCys_C_ in Figure S26. The integrals of ^1^H‐NMR of the CbzCys block in the peptides always decrease after polymerization of the third monomer NPPhe, which may be due to the loss of some self‐polymerized oligocysteines during the precipitation process. After completion of the polymerization, the peptide DMAc solution is precipitated in diethyl ether to obtain the solid peptides. Some self‐polymerized oligocysteines will dissolve into the diethyl ether, leading to the decrease of the integrals within the ^1^H‐NMR. The GPC result shows that Bu‐CbzCys_C_ has an average molecular mass of 2800 and a PDI of 1.5, which also indicates that NNPCbzCys is not suitable for syntheses of polycysteines with large molecular mass and small polydispersity (Figure S27).

The deprotection of S‐carbobenzoxy group can be achieved with a yield of around 85 % yield by a reaction with HBr for 2 h at room temperature. However, the S‐benzyl group can only be removed by reacting with triflic acid at 40 °C.^[17]^ Around 90 % of S‐benzyl group can be removed by reacting with triflic acid at 40 °C.

### Capsule Preparation and Characterization

The preparation of PFD‐filled peptide capsules in this research consisted of two stages. In the first stage, amphiphilic peptide molecules were accumulated at the interfaces of PFD and water to give a metastable emulsion. In the second stage, the cysteine units of peptides were oxidized to form disulfide bridges to solidify the self‐associated structure and to yield a solid peptide membrane. The appearance of a freshly prepared dispersion of PFD‐filled peptide capsules is shown in Figure S28. Initially, the capsule dispersion is well dispersed, but most capsules will settle down during 1 day. However, the capsule dispersion can be re‐dispersed easily by shaking and remains well dispersed for several hours. Such sedimentation will not be relevant during in vivo use, as blood is always in motion so, PFD‐filled capsules are suitable for intravenous application for blood replacement.

**Figure 2:**
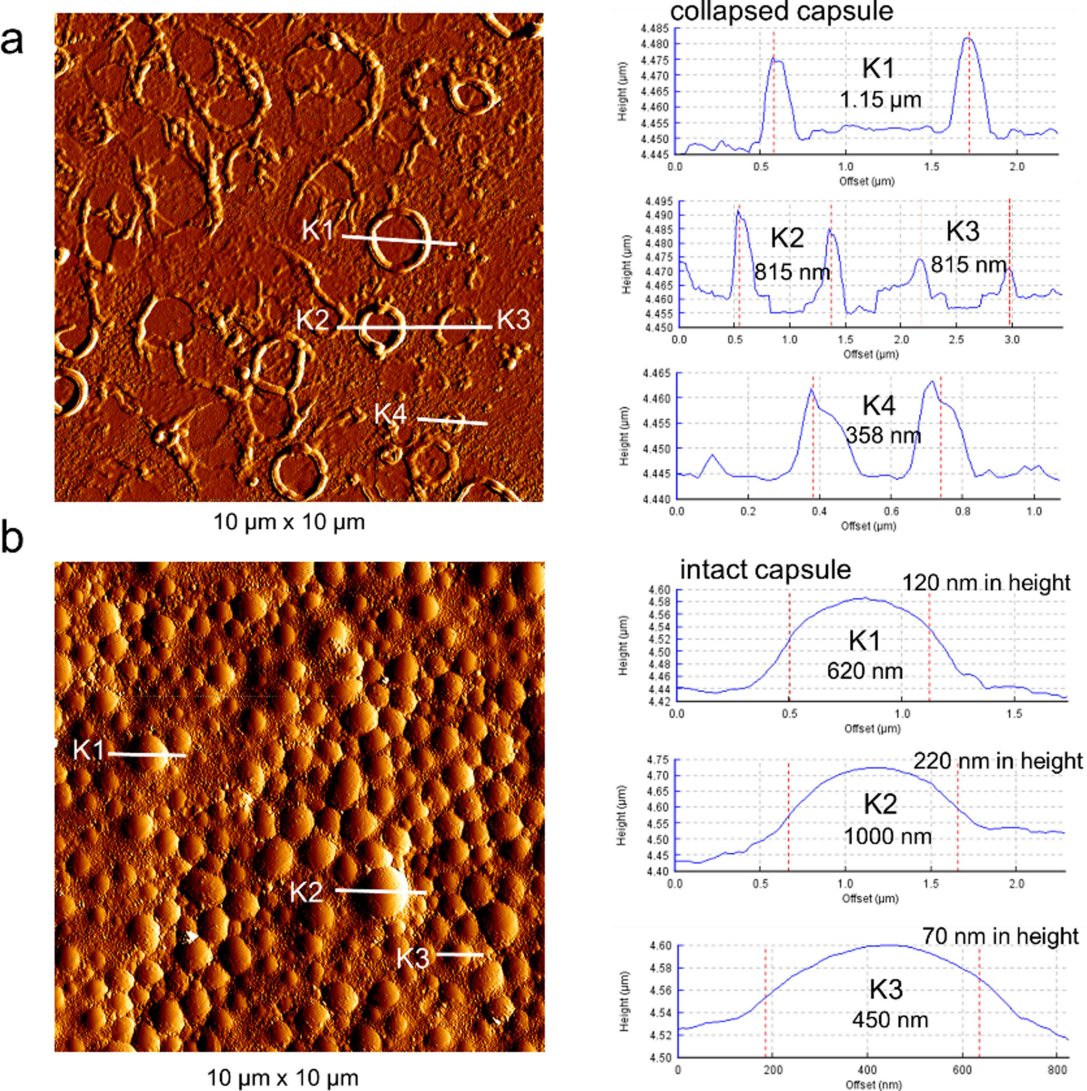
AFM images (left) and height profiles (right) of various PFD‐filled capsules from different triblock peptides. (a) Bu‐Asp_40_‐Cys_5_‐Phe_5_, (b) Bu‐Asp_7_‐Cys_15_‐Phe_2_.

We then analyzed the morphology of dried capsules by AFM. Figure 2 shows AFM images of dried PFD‐filled cysteine‐containing triblock peptide capsules. Figure 2a shows the capsules from Bu‐Asp_40_‐Cys_5_‐Phe_5_. Many regular round hollow structures were observed, which were identified as residual structures from collapsed capsules due to the leakage of PFD. The capsules presented with a diameter that ranged from 300 nm to 1150 nm. The capsule walls exhibited a certain mechanical strength as they retained their round shape even though the PFD was evaporated. The insufficient mechanical strength may be due to the small content of cysteine residues in the peptide. Therefore, triblock peptide with a larger cysteine content were used to encapsulate PFD. Figure 2b shows the dried PFD‐filled Bu‐Asp_7_‐Cys_15_‐Phe_2_ capsules. Most capsules exhibited a diameter that ranged from 300 nm to 1000 nm. Most capsules remained in their spherical shape even after drying, which indicates that their capsule walls exhibit high mechanical strength. Another AFM measurement was performed after the capsule dispersion prepared from Bu‐Asp_7_‐Cys_15_‐Phe_2_ was kept at room temperature for 3 weeks (Figure S29). Most capsules still exhibited intact spherical shapes with diameters that ranged from 300 nm to 1000 nm, which indicates that these capsules can remain stable for at least 3 weeks. We also studied the influence of longer Phe chains length on the morphology of capsules. Figure S30 shows the AFM image of dried PFD filled Bu‐Asp_6_‐Cys_8_‐Phe_8_ capsules. In addition to the spherical capsules and their collapsed counterparts, a large number of very small nanoparticles with diameters around 50 nm were observed, which most likely represent self‐assembled peptide micelles. We also conducted Raman spectroscopy on these capsule dispersions. The Raman spectroscopy confirmed that there existed larger amount of disulfide bridges in these capsule dispersions as shown in Figure S31.

**Figure 3:**
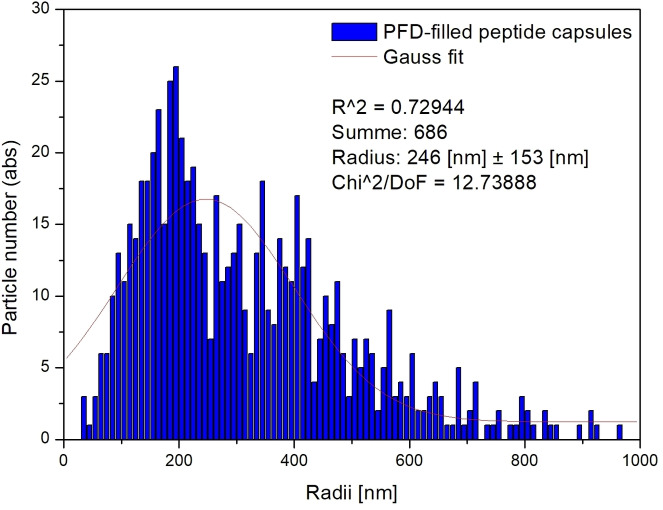
Size distribution of PFD‐filled Bu‐Asp_7_‐Cys_15_‐Phe_2_ capsules as determined by particle tracking.

The size distribution of these capsules was also determined by video microscopic particle tracking.^[18]^ Figure 3 shows the size distribution of PFD‐filled Bu‐Asp_7_‐Cys_15_‐Phe_2_ capsules. It reveals that most of the capsules exhibited diameters between 200 nm and 1000 nm. The size distribution is slightly asymmetric with a tailing towards larger capsules with diameters from 1000 nm to 1700 nm.

**Figure 4:**
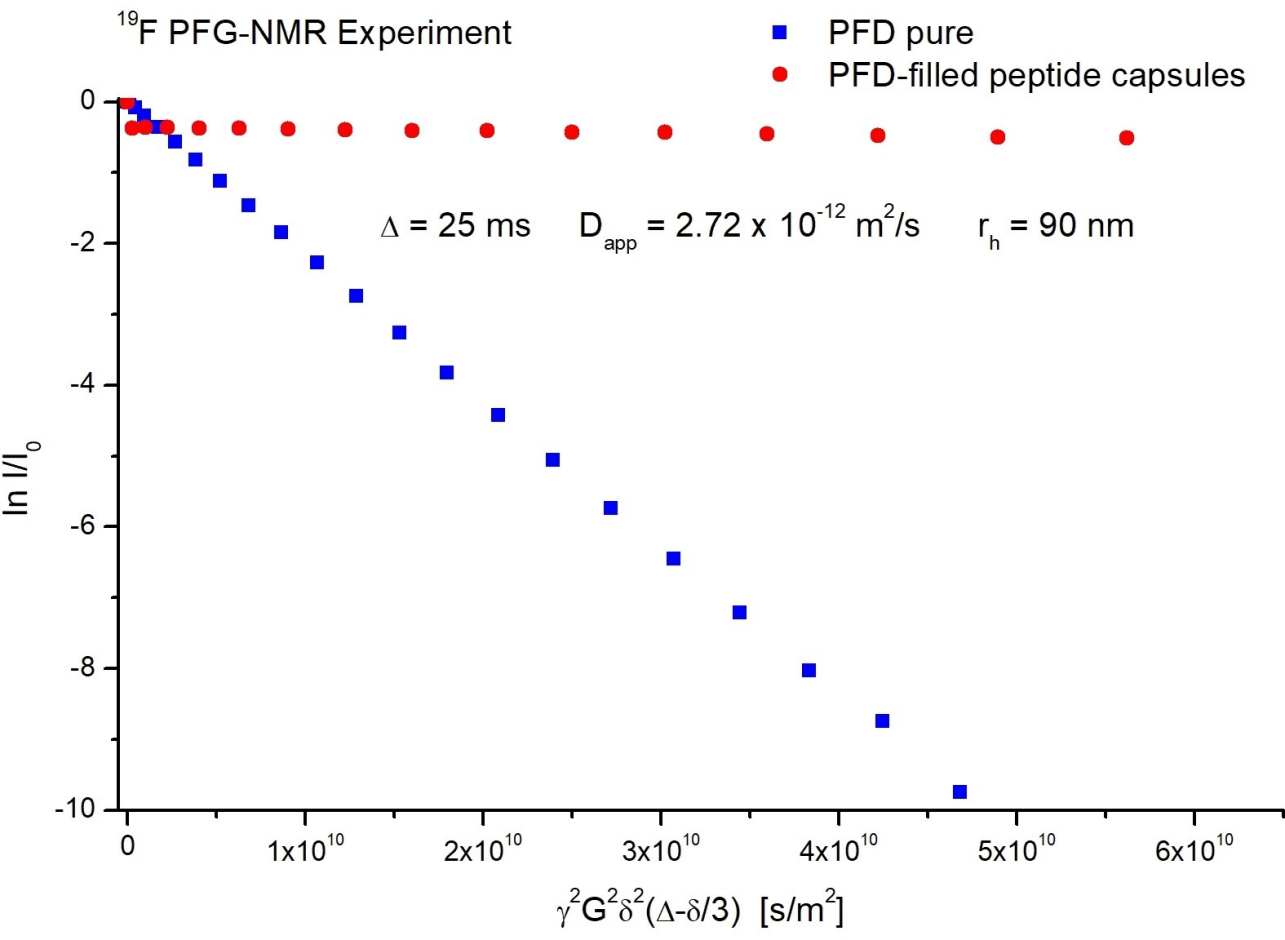
PFG‐NMR echo decay plots for pure liquid PFD and the PFD in a dispersion of PFD‐filled Bu‐Asp_7_‐Cys_15_‐Phe_2_ capsules. The slope obtained for the encapsulated PFD corresponds to a particle diameter of 180 nm. An overall number of 96 measurements in two separate experiments have been averaged for each data point.

The diffusion constant connected to the Brownian motion of these capsules is an important parameter for artificial oxygen carriers, as it can affect the oxygen delivery rate from the dispersion. Therefore, we analyzed the diffusion constant of these capsules by PFG‐NMR, and the result is shown in Figure 4. Our results indicate that the capsules have a diffusion constant of 2.72*10^−12^ m^2^/s. This is about an order of magnitude larger than the self‐diffusion constant connected to the Brownian motion of red blood cells, which means that our capsules can move faster and more efficiently in blood than red blood cells. Therefore, these capsules allow for a quick gas exchange within the various tissue environments. In the meantime, PFD inside the capsules also relies on fast self‐diffusion, which directly supports rapid oxygen absorption and release. PFG NMR data also reveal that these capsules exhibited an average diameter of around 180 nm based on the diffusion constant of the capsules calculated according to the Stokes‐Einstein equation.

**Figure 5:**
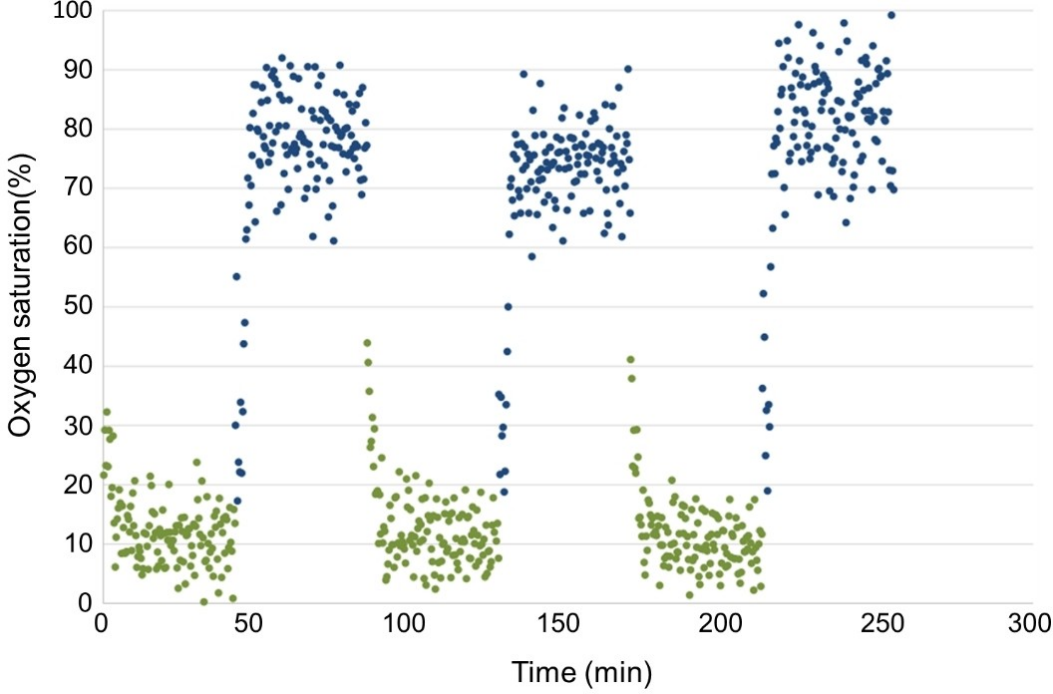
Gas exchange process in a dispersion of PFD‐filled Bu‐Asp_6_‐Cys_8_‐Phe_8_ capsules as determined by the chemical shift of the ^19^F‐NMR signal. The data were observed under sequential purging with O_2_ (blue dots) and with N_2_ (green dots) of the capsule dispersion. Three separate measurements have been averaged for each data point.

To be used as artificial oxygen carriers, the capsule wall should allow for fast gas exchange. Therefore, we analyzed the oxygen uptake and release rate of these capsules by ^19^F‐NMR, the result is shown in Figure 5. The chemical shift of the fluorine nuclei strongly depends on the oxygen concentration due to the paramagnetic influence of the oxygen.^[3]^ The time resolved plot obtained under periodic change of the purging gas between nitrogen and oxygen shows that it took the PFD capsules less than 2 min to equilibrate to the new atmospheric conditions after switching from nitrogen to oxygen or vice versa. After 3 times of nitrogen and oxygen exchange, the capsules still maintained their original oxygen carrying capacity. These results indicate that the capsules allow for fast gas exchange and that the gas exchange is completely reversible. These are important prerequisites for in vivo oxygenation of such capsules in the lung.

**Figure 6:**
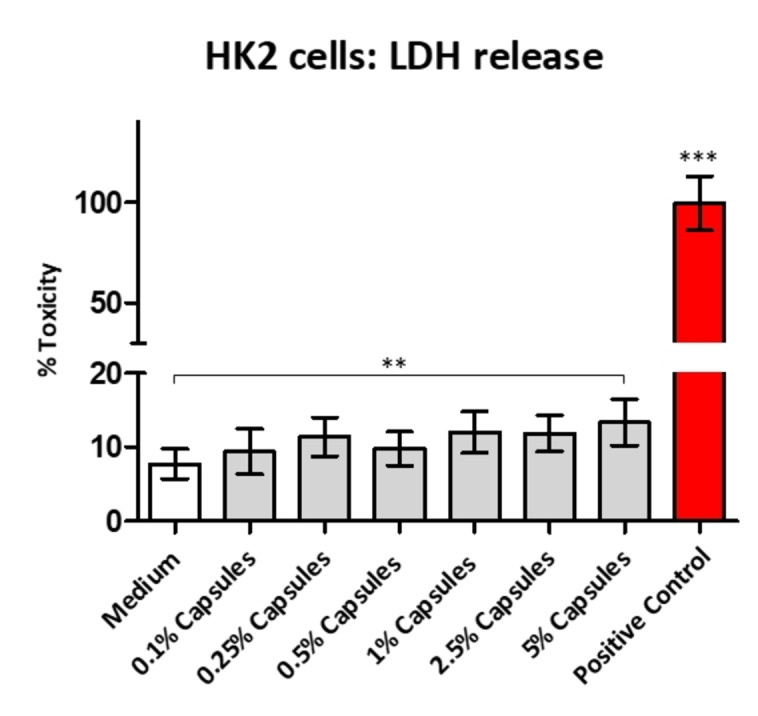
Cytotoxic effects of PFD‐filled capsules on human HK2 cells. To determine the disruption of cellular membrane integrity, LDH analyses was performed. Control cells were incubated for 4 h with DMEM/F‐12 with GlutaMAX (no supplements), whereas treatment groups were incubated for 4 h with nanocapsules at different concentrations (0.1 %, 0.25 %, 0.5 %, 1 %, 2.5 %, 5 %), respectively. The positive control group corresponded to 10 μl Lysis Buffer treatment for 45 min. Values are expressed as means±SD (n=12) from 4 different experiments. t‐test, two‐way ANOVA with Tukey's multiple comparisons test** P<0.05, *** P<0.0001 as compared to medium.

Finally, we determined the cytotoxicity of PFD‐filled peptide capsules by use of the LDH Assay. The LDH Assay result is shown in Figure 6. LDH release is a common marker of cell membrane damage. In our test, the level of LDH leakage from the cells slightly increased after 4 h of treatment with 5 % capsules, whereas at the exposure with 0.1 %–2.5 % capsules, the cells exhibited similar LDH values and kept a high cell viability comparable to control medium.

## Conclusions

We synthesized various cysteine‐containing triblock peptides and successfully prepared corresponding nanocapsules with encapsulated PFD. Most capsules had a suitable diameter range from 200 nm to 1000 nm. These capsules exhibited a certain mechanical strength and allowed for fast gas exchange. The capsules also exhibited little cytotoxicity. These features make the PFD‐filled peptide capsules promising candidates for an application as potential oxygen carriers.

## Conflict of interests

No potential conflict of interest was reported by the authors.

1

## Supporting information

As a service to our authors and readers, this journal provides supporting information supplied by the authors. Such materials are peer reviewed and may be re‐organized for online delivery, but are not copy‐edited or typeset. Technical support issues arising from supporting information (other than missing files) should be addressed to the authors.

Supporting Information

## Data Availability

The data that support the findings of this study are available from the corresponding author upon reasonable request.
