# Supplementary material for: Cross‐linked Triblock Peptide Capsules as Potential Oxygen Carriers
Source: ChemistryOpen. 2024 Mar 12;13(4):e202300282. doi: 10.1002/open.202300282 (PMC11004465; doi:10.1002/open.202300282)
Supplement: Supplementary file 1 — Supporting Information [file OPEN-13-e202300282-s001.pdf]

ChemistryOpen

Supporting Information

Cross-linked Triblock Peptide Capsules as Potential Oxygen Carriers

Huayang Feng,* Jürgen Linders, Miriam Cantore, Jonas Fabrizi, Annika Kirsten, Sascha Myszkowska, Eva Hillen, Florian Uteschil, Sebastian Buchholz, Andrea Hermsen, Maria Davila Garvin, Katja Bettina Ferenz, and Christian Mayer*

Supporting Information

Cross-linked Triblock Peptide Capsules as Potential Oxygen Carriers

Huayang Feng[1]*, Jürgen Linders[1], Miriam Cantore[2], Jonas Fabrizi[3], Annika Kirsten[1], Sascha Myszkowska[1], Eva Hillen[2], Florian Uteschil[4], Sebastian Buchholz[5], Andrea Hermsen[1,6], Maria Davila Garvin[1], Katja Bettina Ferenz[2], Christian Mayer[1]*

[1] Institute for Physical Chemistry, CeNIDE, University of Duisburg-Essen, 45141 Essen, Germany

[2] Institute of Physiology, University Hospital Essen, CeNIDE, University of Duisburg-Essen, 45147 Essen, Germany

[3] Institute of Inorganic Chemistry and Structural Chemistry, Heinrich-Heine-Universität Düsseldorf, 40225 Düsseldorf, Germany

[4] Applied Analytical Chemistry, University of Duisburg-Essen, 45141, Essen, Germany

[5] Institute for Technical Chemistry, University of Duisburg-Essen, 45141 Essen, Germany

[6] Department of Chemistry and ILOC, Niederrhein University of Applied Sciences, 47805 Krefeld, Germany

* CONTACT Huayang Feng: fenghuayang121@gmail.com

Christian Mayer: christian.mayer@uni-due.de

Table S1. Summarized syntheses of Bu-BnAsp$_m$-BnCys$_c$-Phe$_n$.

Synthesis	Initiator	Monomer	Time	Product	Quantity	Yield
1.1	Butylamine (21.9 mg, 73.14, 0.3 mmol)	NNPBnAsp (1.23g, 388, 3.17 mmol, 10.6 eq.)	1 d	Bu-BnAsp$_6$	350 mg	53%
1.2	Bu-BnAsp$_6$ (307 mg, 1300, 0.24 mmol)	NPBnCys (888 mg, 331, 2.7 mmol, 11.3 eq.)	1 d	Bu-BnAsp$_6$-BnCys$_3$	400 mg	48.5%
1.3	Bu-BnAsp$_6$-BnCys$_3$ (386.7 mg, 1880, 0.206 mmol)	NPBnCys (773 mg, 331, 2.3 mmol, 11.3 eq.)	3 d	Bu-BnAsp$_6$-BnCys$_8$	582 mg	69.5%
1.4	Bu-BnAsp$_6$-BnCys$_8$(520 mg, 2850, 0.182 mmol)	NPPhe (627 mg, 285, 2.2 mmol, 11 eq.)	3 d	Bu-BnAsp$_6$-BnCys$_8$-PPhe$_8$	740 mg	87.8%
2.1	Butylamine (28.7 mg, 73.14, 0.393 mmol)	NNPBnAsp (3.05 g, 388, 7.86 mmol, 20 eq.)	1 d	Bu-BnAsp$_{13}$	857 mg	53.2%
2.2	Bu-BnAsp$_{13}$ (400 mg, 2740, 0.146 mmol)	NPBnCys (520 mg, 331, 1.57 mmol, 10.8 eq.)	1 d	Bu-BnAsp$_{13}$-BnCys$_6$	520 mg	74.0%
2.3	Bu-BnAsp$_{13}$-BnCys$_6$ (490 mg, 3900, 0.126 mmol)	NPPhe (378 mg, 285, 1.33 mmol, 10.6 eq.)	1 d	Bu-BnAsp$_{13}$-BnCys$_6$-Phe$_7$	605 mg	88.3%
3.1	Butylamine (34.5 mg, 73.14, 0.472 mmol)	NPBnAsp (2 g, 338, 5.9 mmol, 12.5 eq.)	3 d	Bu-BnAsp$_7$	861.4 mg	71.0 %

3.2	Bu-BnAsp$_7$ (230 mg, 1500, 0.147 mmol)	NPBnCys (730 mg, 331, 2.2 mmol, 15 eq.)	2 d	Bu-BnAsp$_7$-BnCys$_{15}$	465 mg	70.8%
3.3	Bu-BnAsp$_7$-BnCys$_{15}$ (370 mg, 4400, 0.084 mmol)	NPPhe (65 mg, 285, 0.23 mmol, 2.7 eq.)	2 d	Bu-BnAsp$_7$-BnCys$_{15}$-Phe$_2$	330 mg	81.7%

Table S2. Summarized syntheses of Bu-BnAsp$_m$-CbzCys$_c$-Phe$_n$.

Synthesis	Initiator	Monomer	Temperature	Product	Quantity	Yield
1.1	Butylamine (13.1 mg, 73.14, 0.179 mmol)	NPBnAsp (3.03 g, 338, 8.96 mmol, 50 eq.)	60 °C	Bu-BnAsp$_{31}$	1.02 g	55.5 %
1.2	Bu-BnAsp$_{31}$ (500 mg, 6400, 0.078 mmol)	NNPCBzCys (1.35 g, 420, 2.5 mmol, 32 eq.)	60 °C	Bu-BnAsp$_{31}$-CbzCys$_5$	700 mg	55.6 %
1.3.1	Bu-BnAsp$_{31}$-CbzCys$_5$ (300 mg, 7600, 0.039 mmol)	NPPhe (111.1 mg, 285, 0.39 mmol, 10 eq.)	60 °C	Bu-BnAsp$_{31}$-CbzCys$_5$	230 mg	64.4 %
1.3.2	Bu-BnAsp$_{31}$-CbzCys$_5$ (200 mg, 7600, 0.026 mmol)	NPPhe (300 mg, 285, 1.05 mmol, 40 eq.)	60 °C	Bu-BnAsp$_{31}$-CbzCys$_5$-Phe$_4$	230 mg	64.8 %
2.1	Butylamine (34.7 mg, 73.14, 0.474 mmol)	NPBnAsp (8 g, 338, 23.7 mmol, 50 eq.)	60 °C	Bu-BnAsp$_{40}$	3.85 g	79.3 %
2.2	Bu-BnAsp$_{40}$ (514 mg, 8250, 0.062 mmol)	NNPCbzCys (840 mg, 420, 2 mmol, 33 eq.)	30 °C	Bu-BnAsp$_{40}$-CbzCys$_{10}$	688 mg	68.9 %
2.3	Bu-BnAsp$_{40}$-CbzCys$_{10}$ (305 mg, 10600, 0.029 mmol)	NNPPhe (280 mg, 330, 0.85 mmol, 28 eq.)	30 °C	Bu-BnAsp$_{40}$-CbzCys$_{10}$-Phe$_5$	330 mg	76.7 %

Table S3 Summarized de-protection reaction of triblock peptide.

Synthesis	Protected peptide	Solvent	Acid	Product	Quantity
1	Bu-BnAsp$_{31}$-CbzCys$_5$-Phe$_4$ (140 mg, 8200, 17 μmol)	3 mL TFA	1 mL 33% HBr/ CH_3COOH	Bu-Asp$_{31}$-Cys$_5$-Phe$_4$	100 mg, 4700, 21 μmol
2	Bu-BnAsp$_{40}$-CbzCys$_5$-Phe$_5$ (195 mg, 10200 , 19.1 μmol)	2 mL TFA	1 mL 33% HBr/ CH_3COOH	Bu-Asp$_{40}$-Cys$_{10}$-Phe$_5$	-
3	Bu-BnAsp$_6$-BnCys$_8$-Phe$_8$(66 mg, 4000, 16.5 μmol)	5 mL CH_2Cl_2	CF_3SO_3H (1 mL, 11.3 mmol/mL, 11.3 mmol)	Bu-Asp$_6$-Cys$_8$-Phe$_8$	43 mg, 2670, 16 μmol
4	Bu-BnAsp$_{13}$-BnCys$_6$-Phe$_7$ (191.9 mg, 4920, 0.039 mmol)	5 mL CH_2Cl_2	CF_3SO_3H (1 mL, 11.3 mmol/mL, 11.3 mmol)	Bu-Asp$_{13}$-Cys$_6$-Phe$_7$	140 mg, 3210, 43 μmol
5	Bu-BnAsp$_7$-BnCys$_{15}$-Phe$_2$ (250 mg, 4700, 0.053 mmol)	3 mL TFA	CF_3SO_3H (1 mL, 11.3 mmol/mL, 11.3 mmol)	Bu-Asp$_7$-Cys$_{15}$-Phe$_2$	280 mg, 2700, 104 μmol

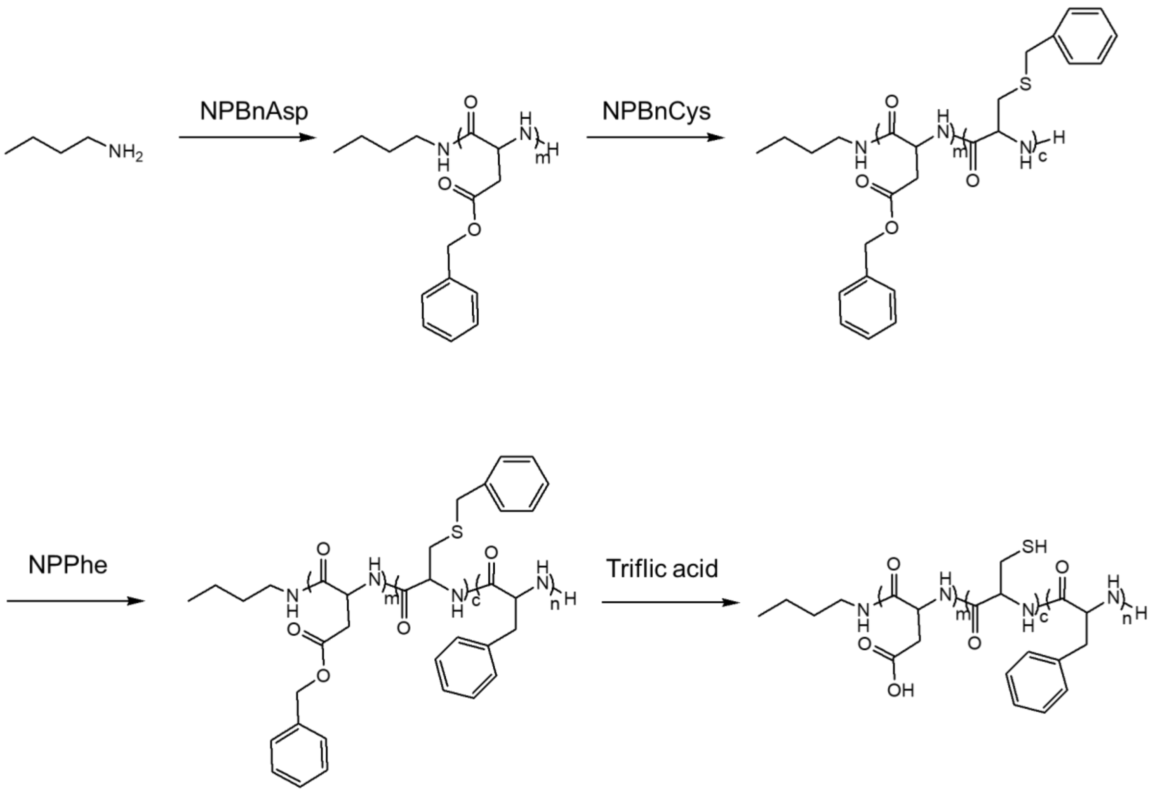

Figure S1. Synthetic route of triblock peptide using NPBnCys as monomer and n-butylamine as initiator.

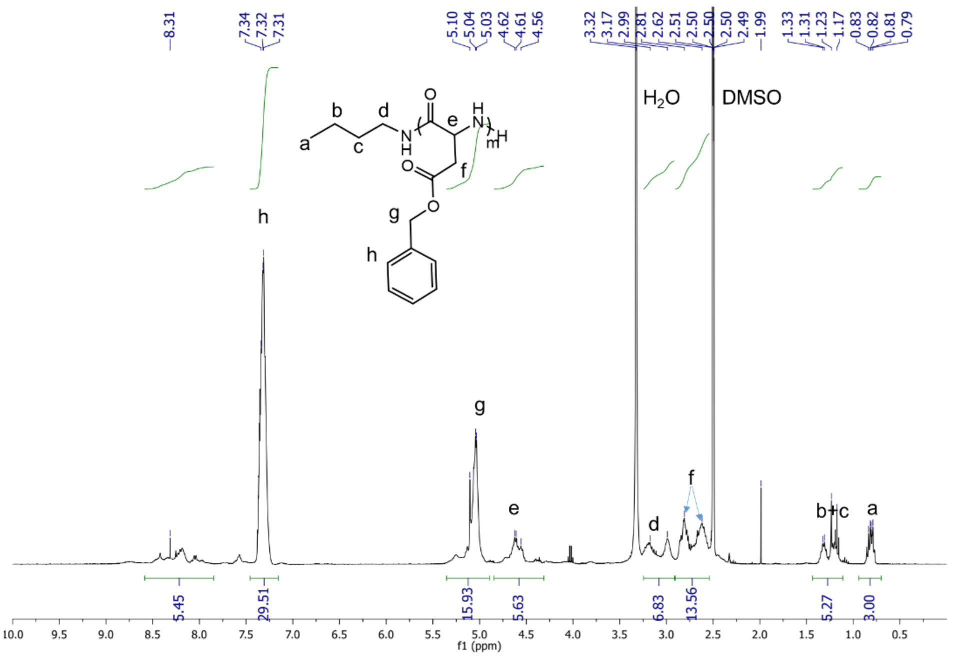

Figure S2. ^1H NMR spectrum of Bu-BnAsp$_6$ (solvent: DMSO-d_6).

According to the integrals of the signals a and h, the average number of BnAsp units is approximately 6.

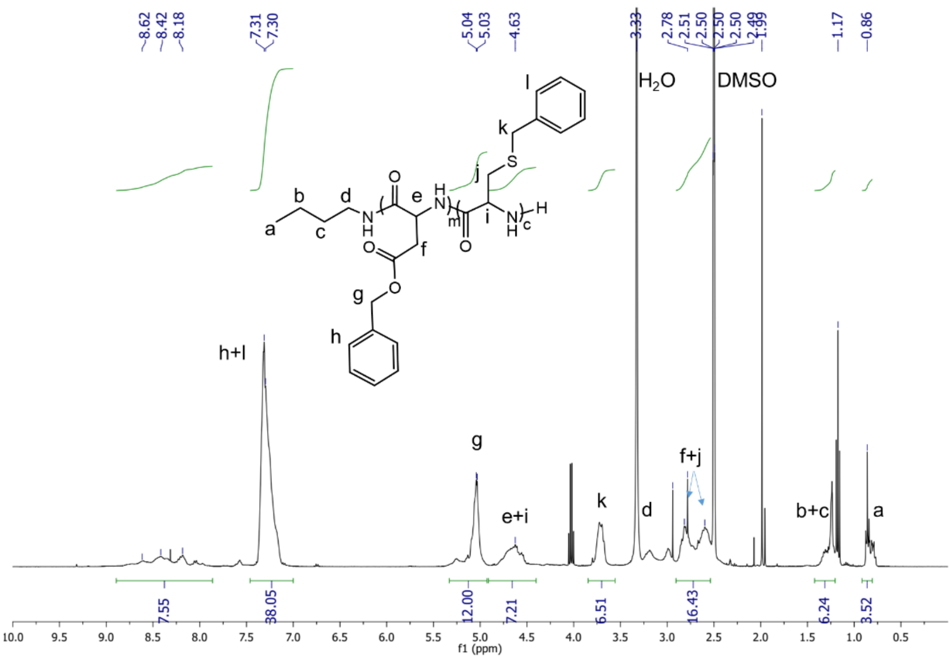

Figure S3 ^{1}H NMR spectrum of Bu-BnAsp$_6$-BnCys$_3$ (solvent: DMSO-d$_6$).

According to the integrals of the signals g and j, the average number of BnCys units is approximately 3.

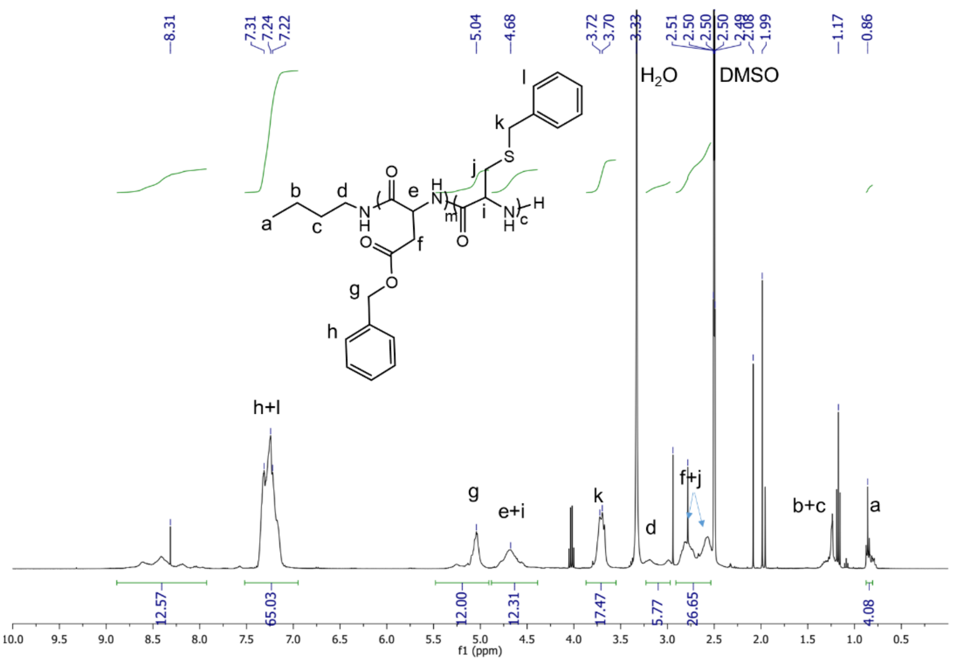

Figure S4. ^{1}H NMR spectrum of Bu-BnAsp$_6$-BnCys$_8$ (solvent: DMSO-d$_6$).

According to the integrals of the signals g and j, the average number of BnCys units is approximately 8. The polymerization of NPBnCys was repeated another time due to the low DP of first polymerization.

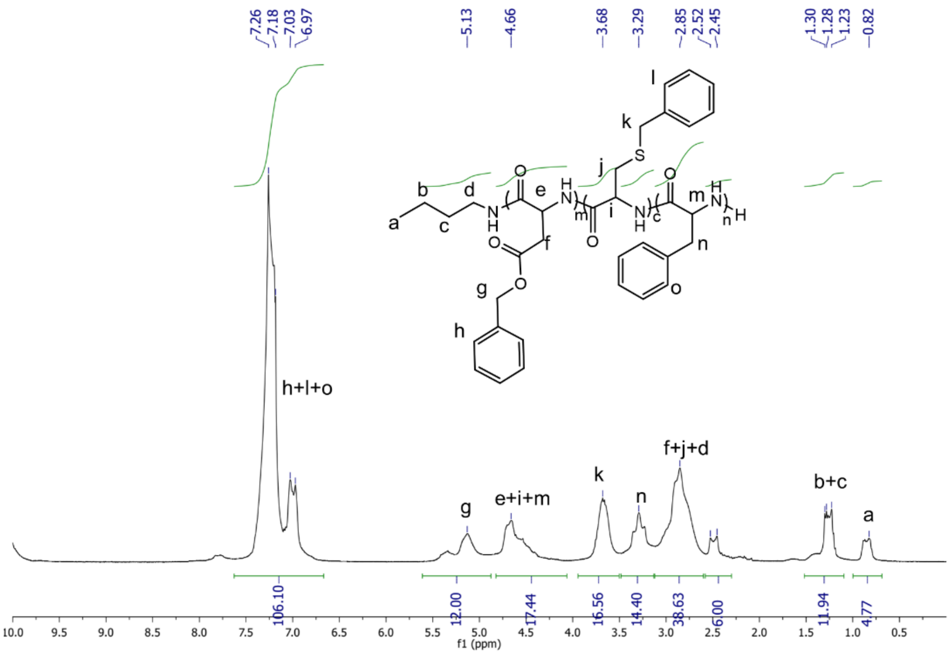

Figure S5. ^{1}H NMR spectrum of Bu-$BnAsp_6$-$BnCys_8$-Phe_8 (solvent: TFA-d_1: $CDCl_3$= 1:1).

According to the integrals of the signals g and k, the average number of Phe units is approximately 8.

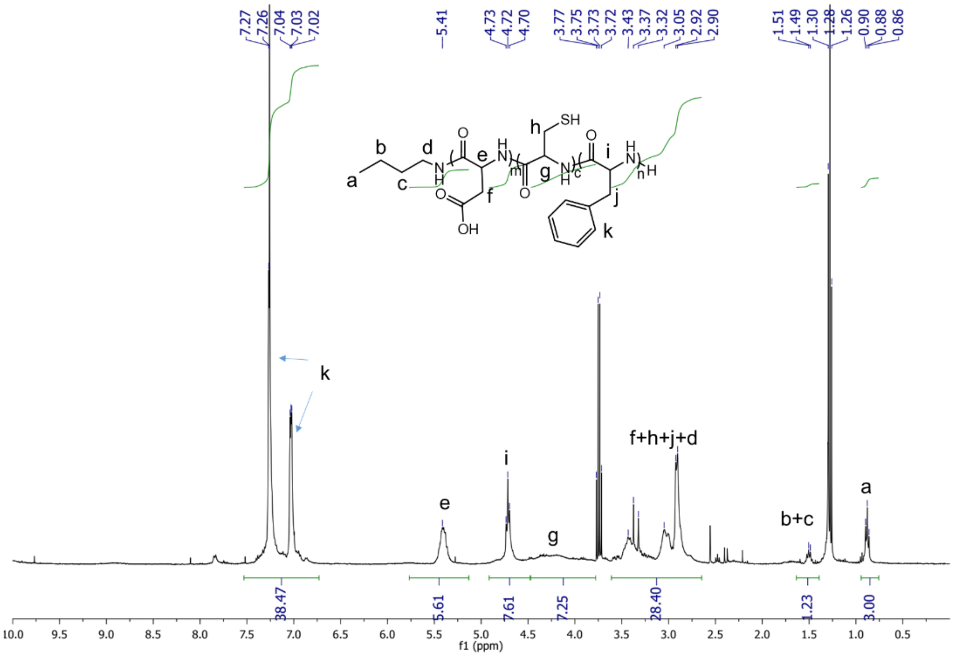

Figure S6. ^{1}H NMR spectrum of Bu-Asp_6-Cys_8-Phe_8 (solvent: TFA-d_1: $CDCl_3$= 1:1).

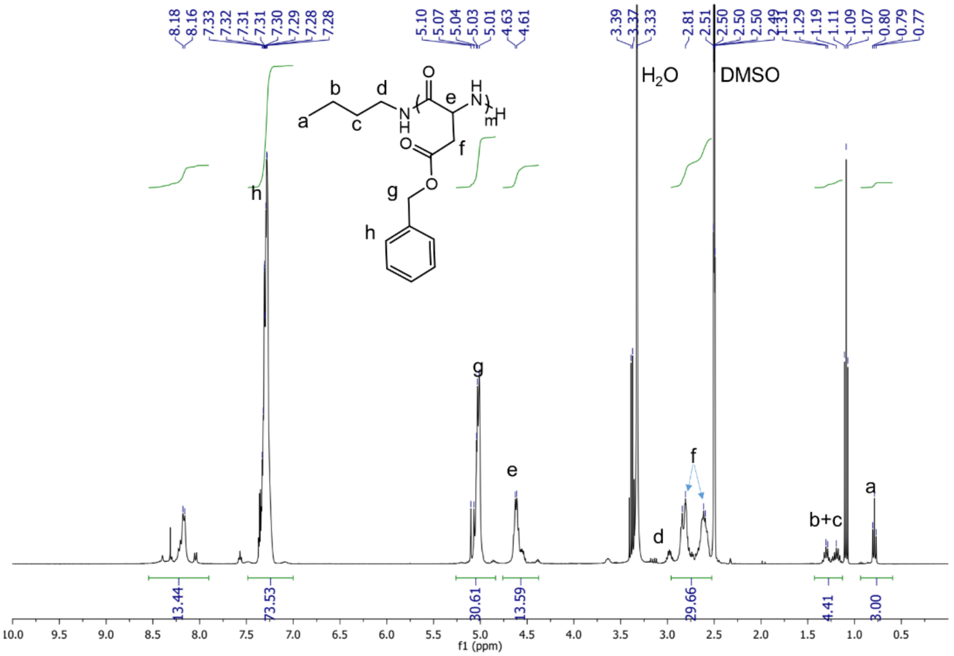

Figure S7. ^{1}H NMR spectrum of Bu-BnAsp$_6$ (solvent: DMSO-d$_6$).

According to the integrals of the signals a and h, the average number of BnAsp units is approximately 13.

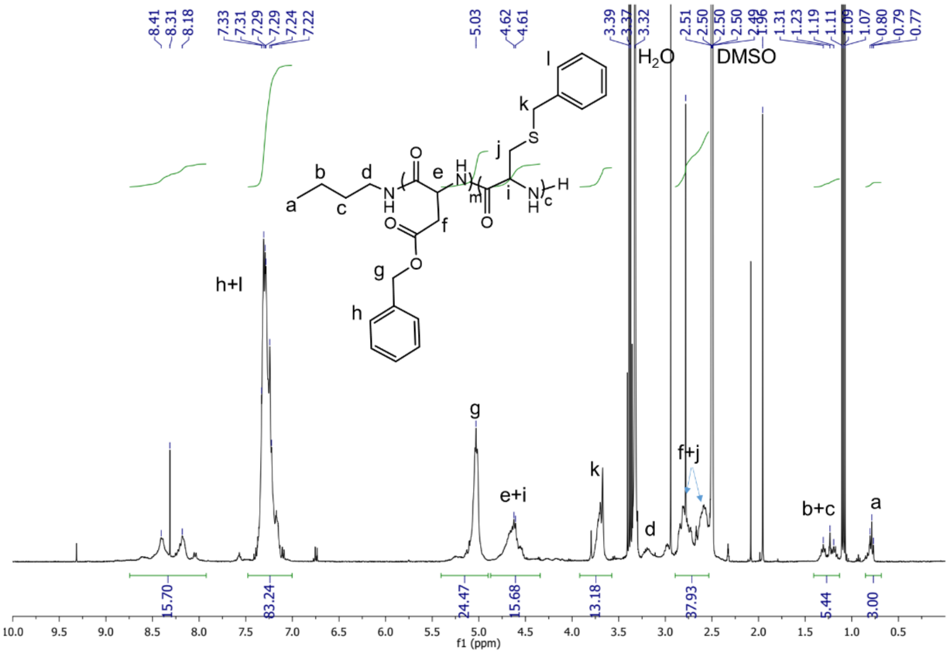

Figure S8. ^{1}H NMR spectrum of Bu-BnAsp$_6$-BnCys$_3$ (solvent: DMSO-d$_6$).

According to the integrals of the signals a and k, the average number of BnCys units is approximately 6.

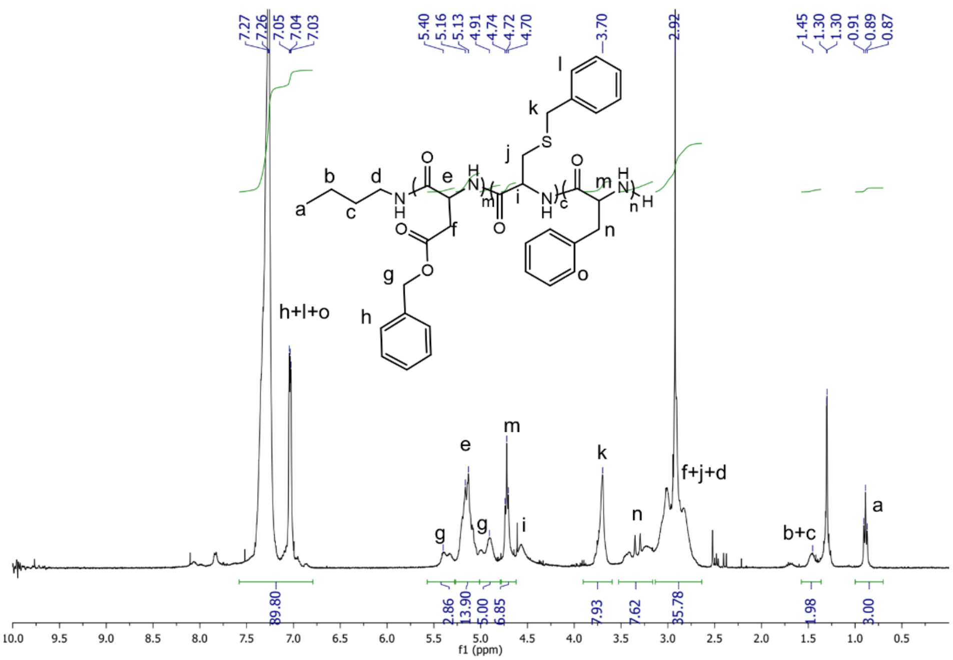

Figure S9. ^{1}H NMR spectrum of Bu-BnAsp$_{13}$-BnCys$_{6}$-Phe$_{7}$ (solvent: TFD-d$_{1}$ and $CDCl_3$).

According to the integrals of the signals a and m, the average number of Phe units is approximately 7.

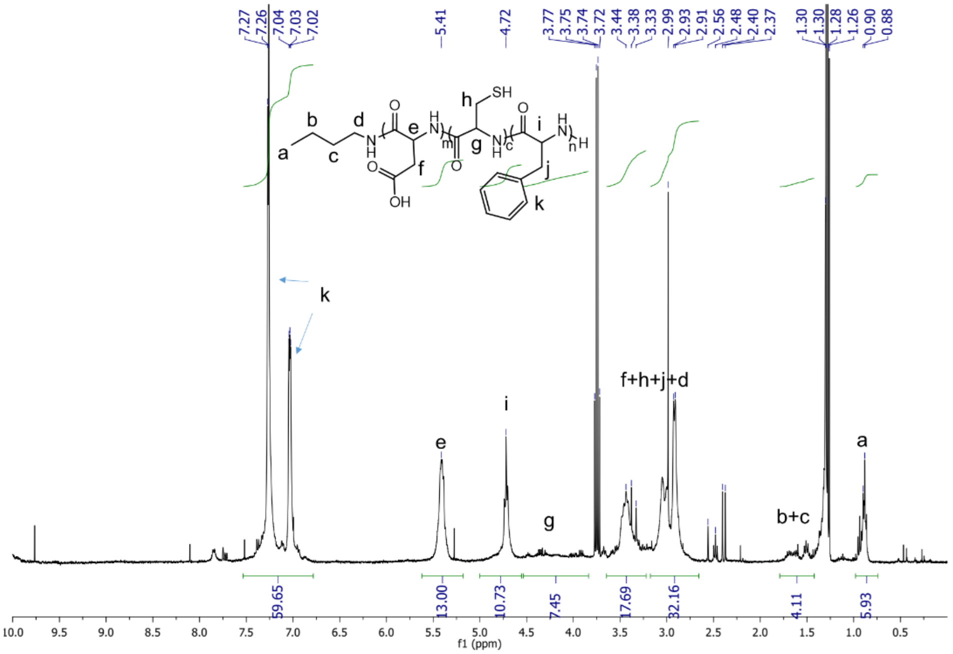

Figure S10. ^{1}H NMR spectrum of Bu-Asp_{13}-Cys_6-Phe_7 (solvent: TFA-d_1: $CDCl_3$= 1:1).

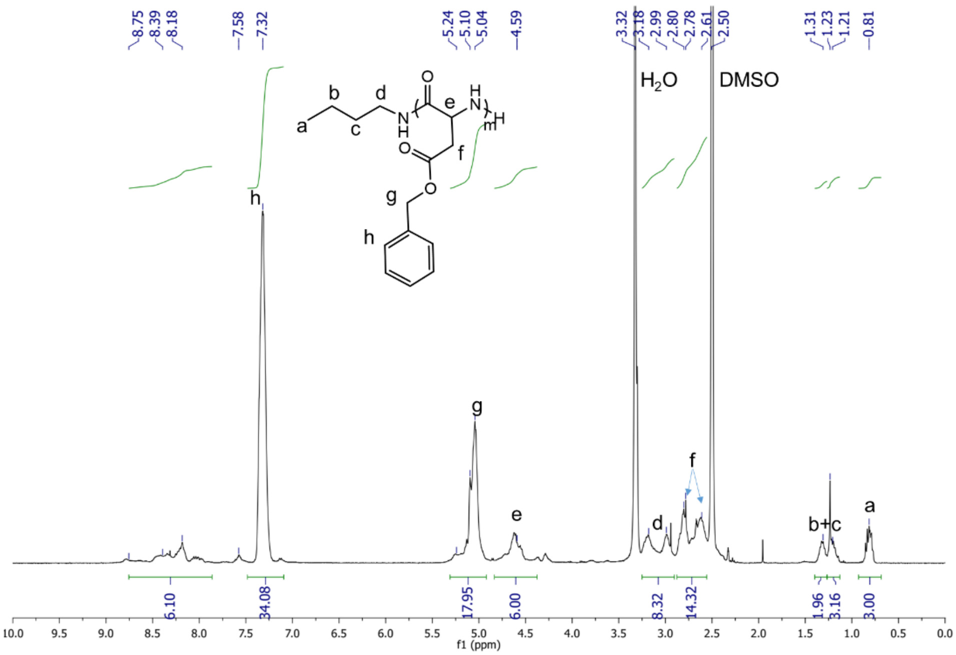

Figure S11 ^{1}H NMR spectrum of Bu-BnAsp$_7$ (solvent: DMSO-d$_6$).

According to the integrals of the signals a and h, the average number of BnAsp units is approximately 7.

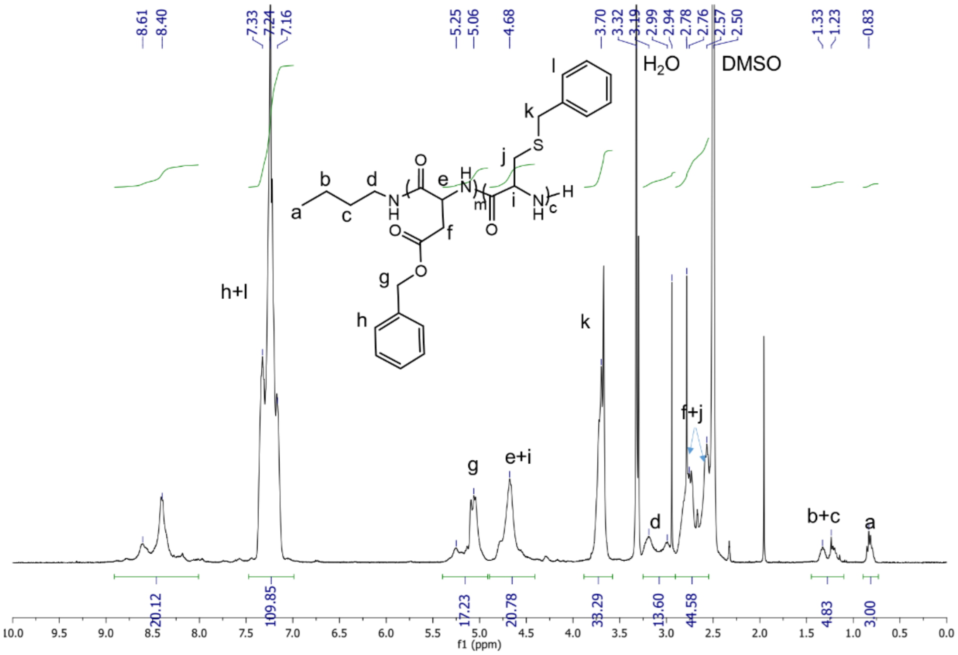

Figure S12 ^{1}H NMR spectrum of Bu-BnAsp$_7$-BnCys$_{15}$ (solvent: DMSO-d$_6$).

According to the integrals of the signals a and h+l, the average number of BnCys units is approximately 15.

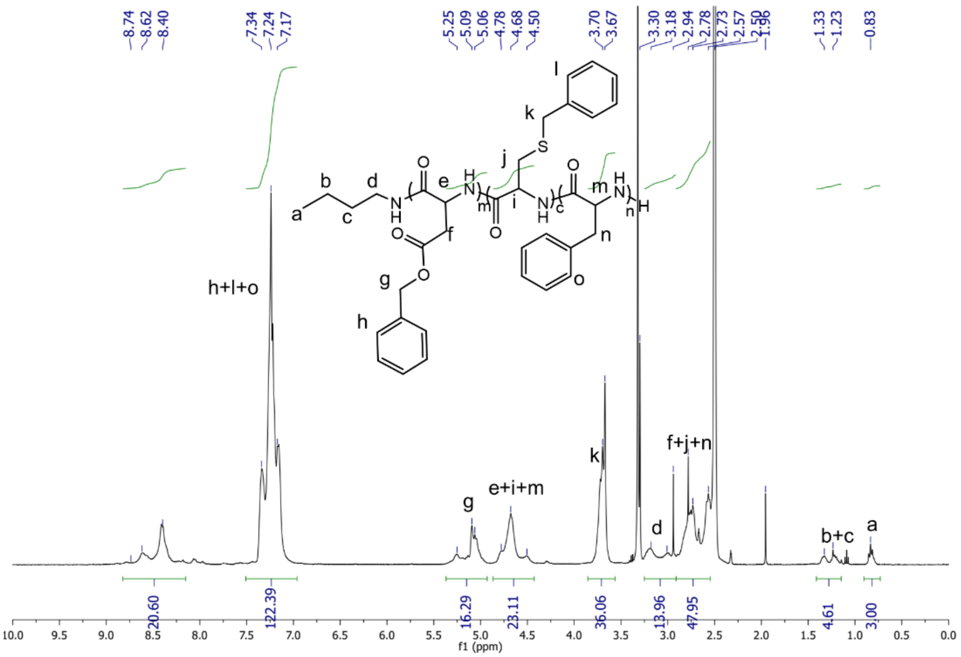

Figure S13 ^{1}H NMR spectrum of Bu-BnAsp$_7$-BnCys$_{15}$-Phe$_2$ (solvent: TFD-d$_1$ and $CDCl_3$).

According to the integrals of the signals a and h+l+o, the average number of Phe units is approximately 2.

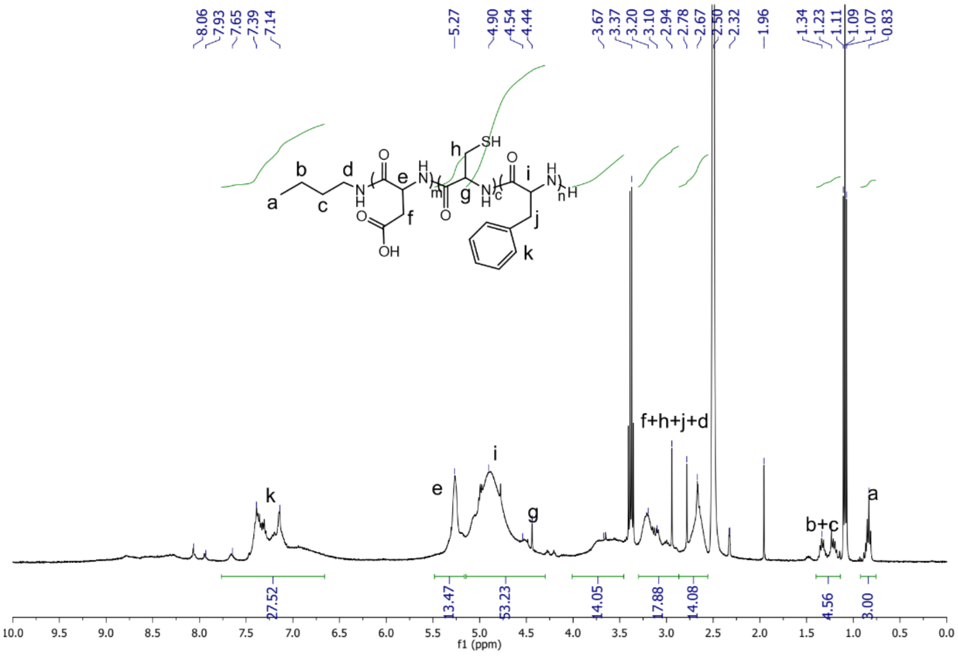

Figure S14 ^{1}H NMR spectrum of Bu-Asp$_7$-Cys$_{15}$-Phe$_2$ (solvent: DMSO-d$_6$).

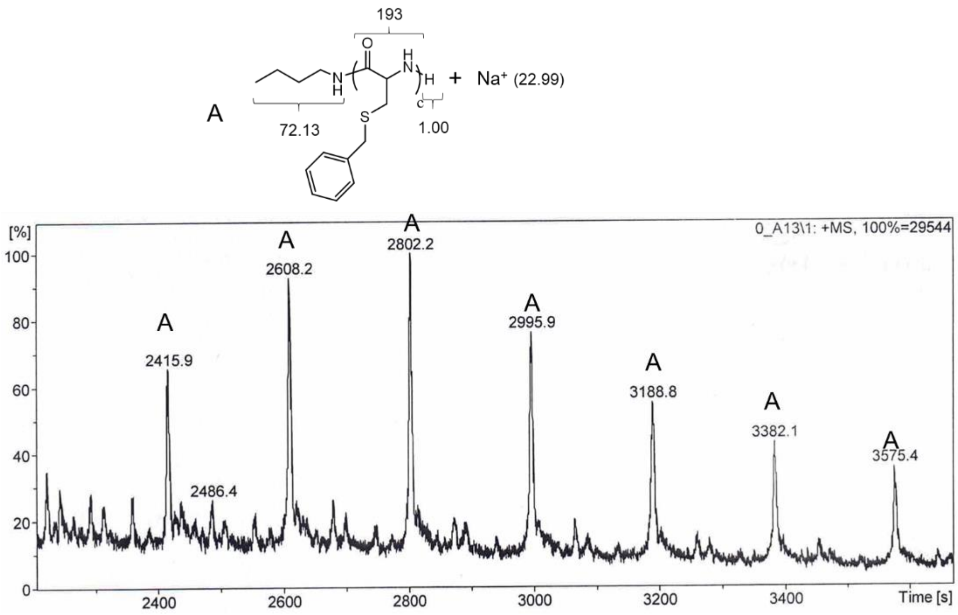

Figure S15. MALDI-TOF mass spectrum of Bu-BnCys$_{17}$.

Mono-block Bu-BnCys$_{17}$ was synthesized in order to study its terminal structure. Only one series of signals which is marked as series-A was observed in the spectrum, and it corresponds to polymers with mass values of around 2416, 2608, 2802, 2996, 3189 and 3382. These signals are regularly located with a spacing of about 193 Da, which is equal to the formula mass of the repeating unit of Bu-BnCys$_c$. The molecular weight of peptide terminal was calculated as around 70. These mass values are consistent with the polymer structure which has a butylamine-derived initiating end and an amino group at the propagating end.

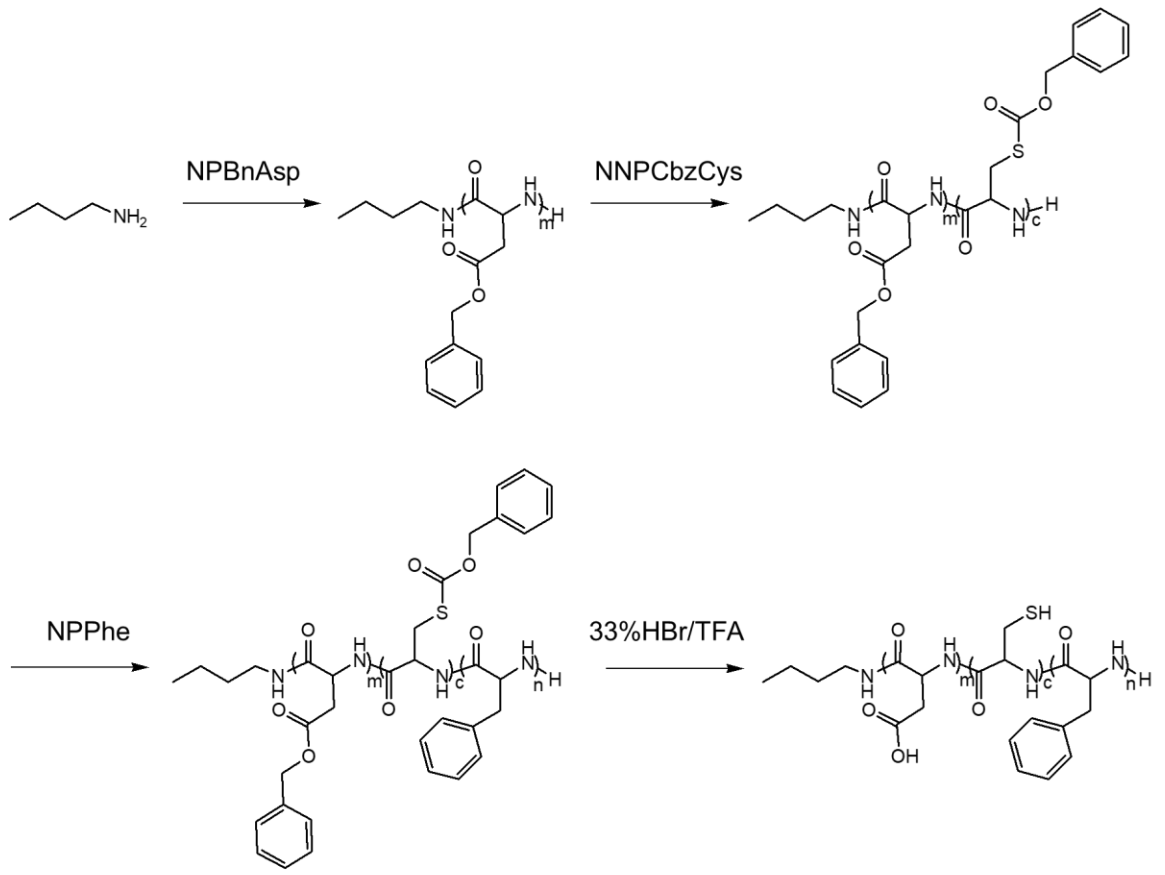

Figure S16. Synthetic route of triblock peptide using NNPCbzCys as monomer and n-butylamine as initiator.

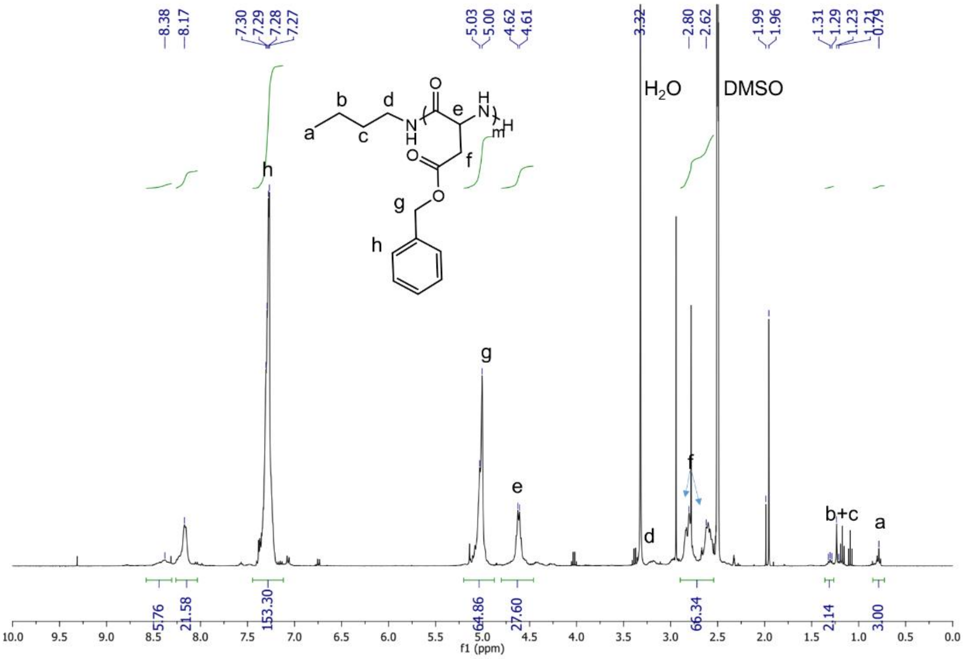

Figure S17. ^{1}H NMR spectrum of Bu-BnAsp$_{31}$ (solvent: DMSO-d$_6$).

According to the integrals of the signals a and h, the average number of Asp units is approximately 31.

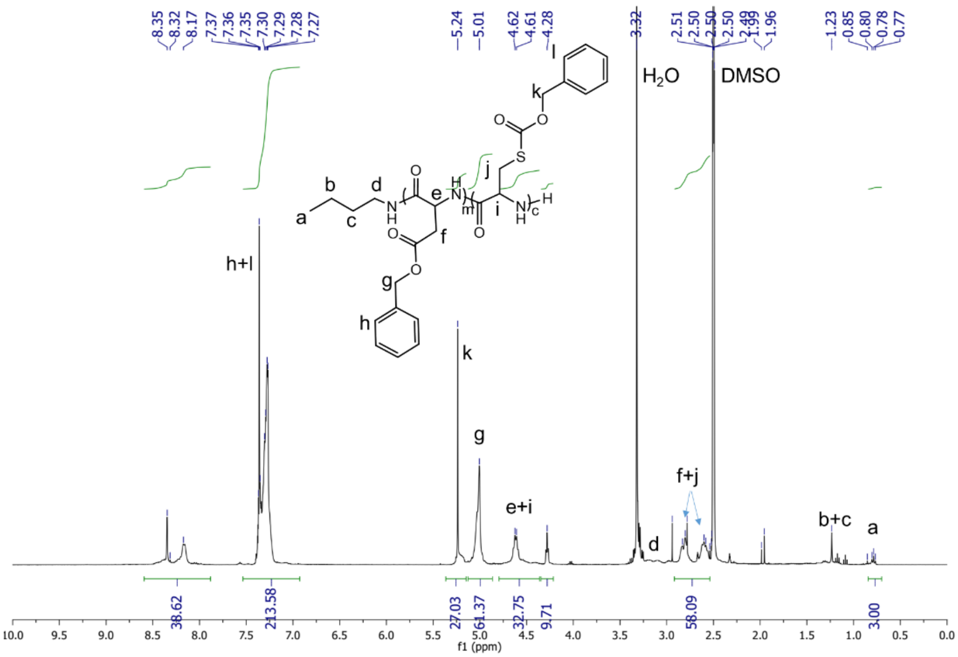

Figure S18. ^{1}H NMR spectrum of Bu-BnAsp$_{31}$-CbzCys$_{14}$ (solvent: DMSO-d$_6$).

According to the integrals of the signals a and k, the average number of Cys units is approximately 14.

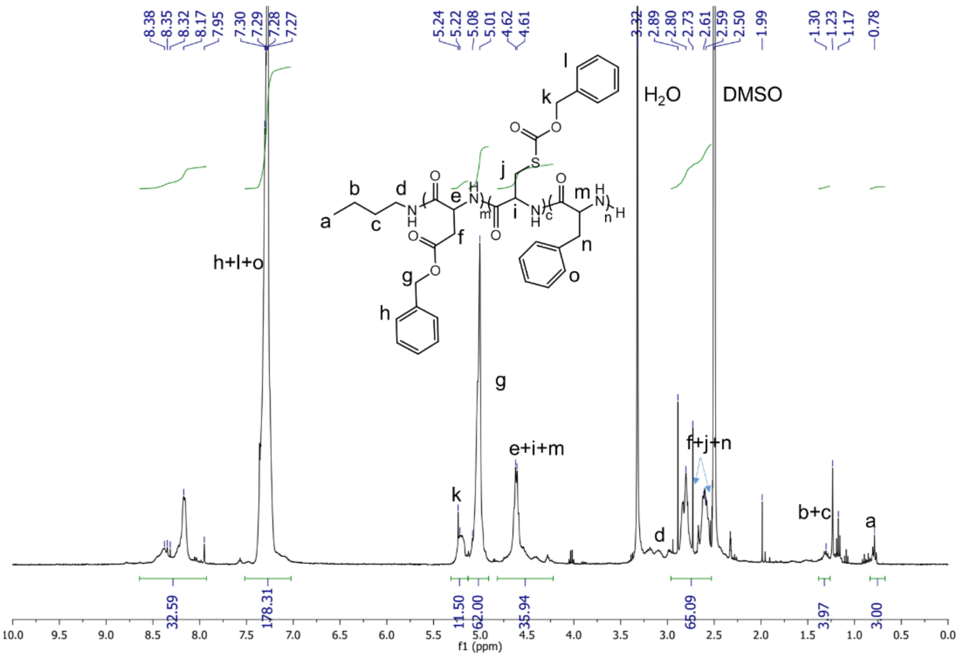

Figure S19. ^{1}H NMR spectrum of Bu-BnAsp$_{31}$-CbzCys$_{6}$-Phe$_{n}$ (solvent: DMSO-d$_{6}$)

The integral for k decreased from 27 to 11.5, possibly due to the loss of self-polymerized oligo-CbzCys during the precipitation process.

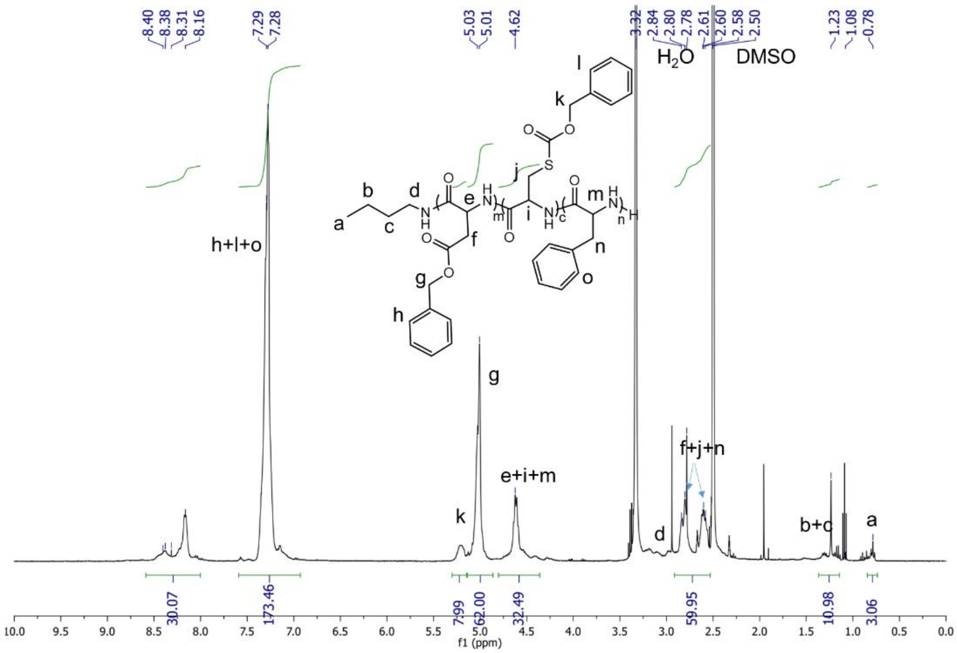

Figure S20. ^{1}H NMR spectrum of Bu-BnAsp$_{31}$-CbzCys$_4$-Phe$_n$ (solvent: DMSO-d$_6$)

Again, the integral for k decreased from 11.5 to 7.99. Therefore, the average number for CbzCys in the peptides should be around 4. The average number of Phe units was determined in the deprotected tri-block peptide.

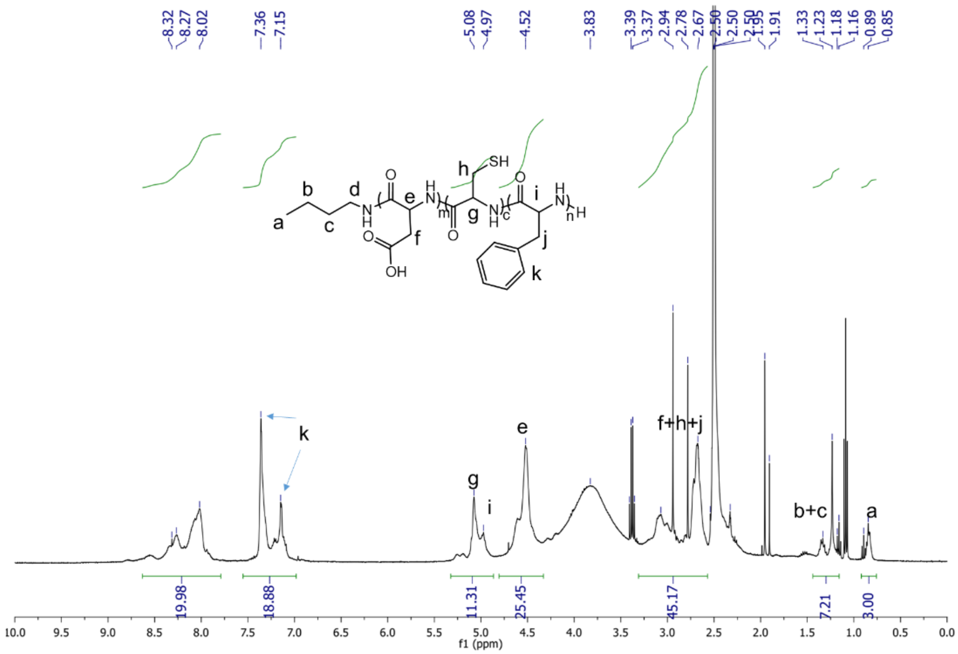

Figure S21. ^{1}H NMR spectrum of Bu-Asp$_{31}$-Cys$_{4}$-Phe$_{3}$ (solvent: DMSO-d$_{6}$).

According to the integrals of the signals a and k, the average number of Phe units is approximately 3.

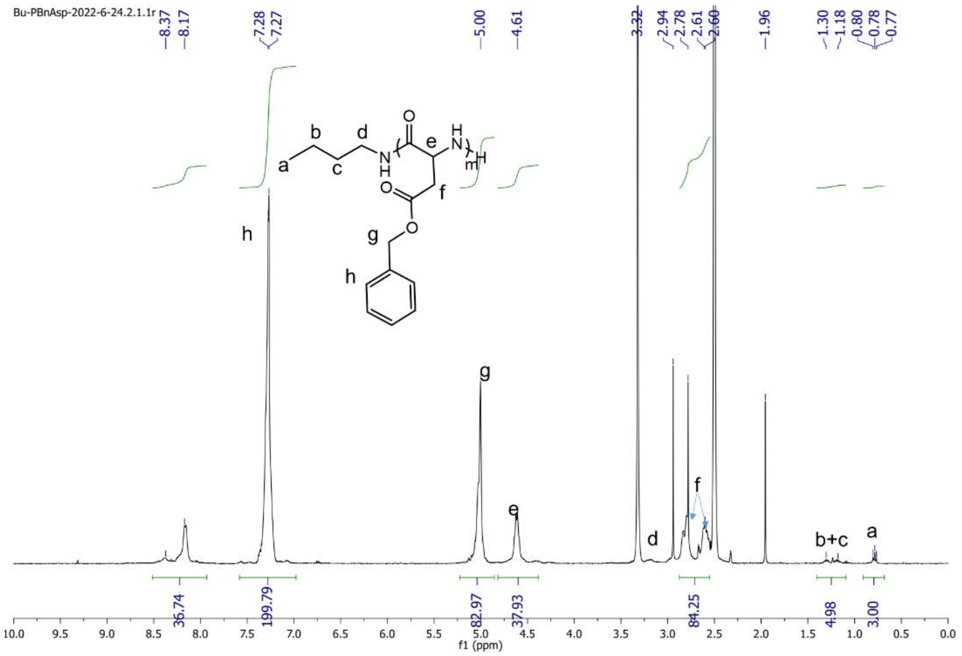

Figure S22. ^{1}H NMR spectrum of Bu-BnAsp$_{40}$ (solvent: DMSO-d_6).

According to the integrals of the signals a and g, the average number of Asp units is approximately 40.

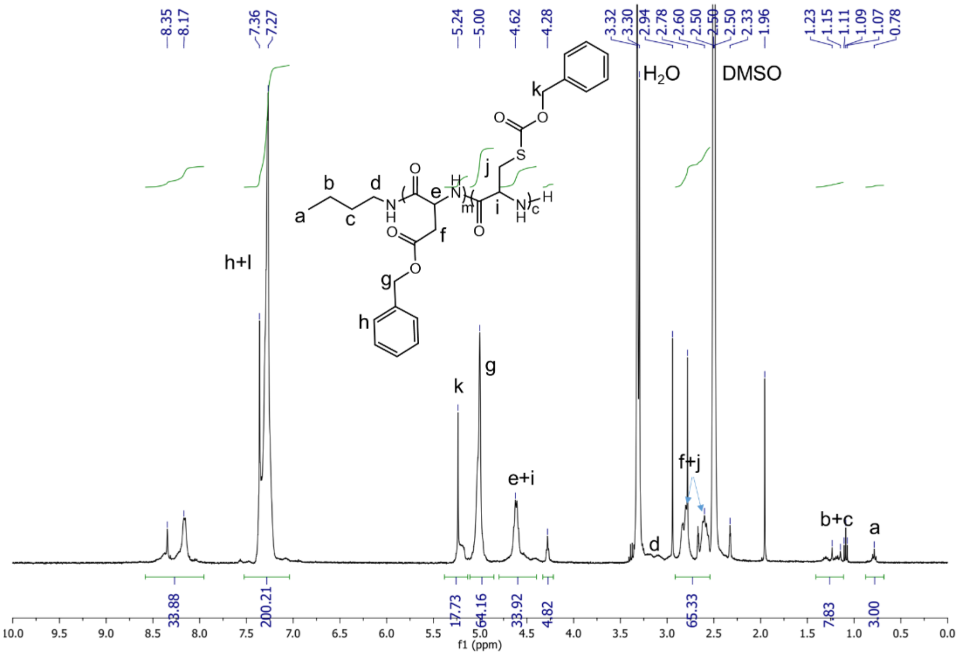

Figure S23. ^{1}H NMR spectrum of Bu-BnAsp$_{40}$-CbzCys$_{9}$ (solvent: DMSO-d_6).

According to the integrals of the signals a and k, the average number of Cys units is approximately 9.

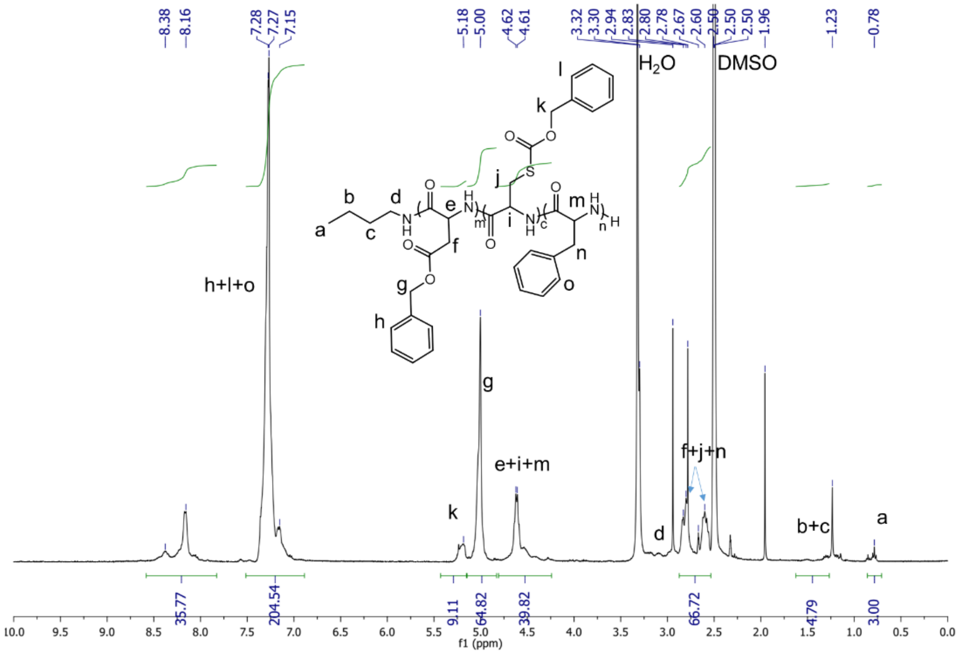

Figure S24. ^{1}H NMR spectrum of Bu-BnAsp$_{40}$-CbzCys$_5$-Phe$_n$ (solvent: DMSO-d$_6$).

Again, the integrals for CbzCys block decreases from 17.73 to 9.11, possibly due to the loss of self-polymerized oligo-CbzCys during the precipitation process. Therefore, the average number for CbzCys in the peptides should be around 5.

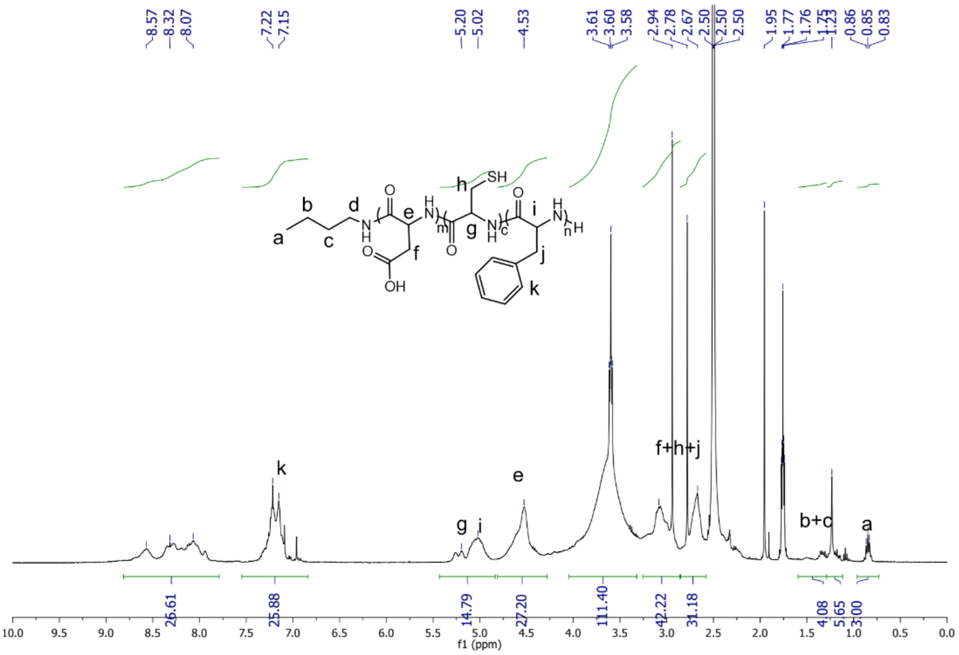

Figure S25 ^{1}H NMR spectrum of Bu-Asp$_{40}$-Cys$_{5}$-Phe$_{5}$ (solvent: DMSO-d_6).

According to the integrals of the signals a and k, the average number of Phe units is approximately 5.

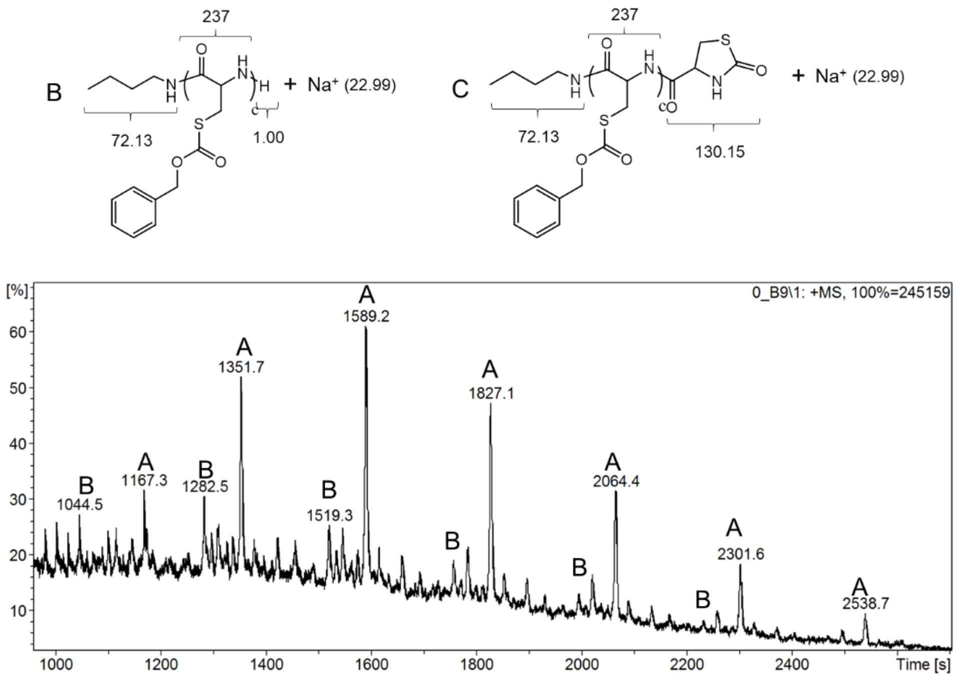

Figure S26. MALDI-TOF mass spectrum of Bu-CbzCys$_{C}$.

Mono-block Bu-CbzCys$_{17}$ was synthesized in order to study its terminal structure. It seemed like there are several series of signals in the spectrum, and series-A signals was much stronger than the other series of signals. Series-A signals correspond to polymers with mass values of about 1353, 1591, 1828, 2065 and 2303. These signals are regularly located with a spacing of around 237 Da, which is equal to the formula mass of the repeating unit of Bu-CbzCys$_{C}$. If there is no cleavage between protecting groups and amino acid residues, the molecular weight of peptide terminal (series-A) was calculated as around 45 or around 282, which is not in agreement with the two possible terminal structure (B and C) as shown in Figure S26. If one protecting group was removed during the measurement, the molecular weight of peptide terminal (series-A) was calculated as around 180 or around 417, which is still not in agreement with the two possible terminal structure (B and C) as shown in Figure S26. Some unknown intramolecular termination reaction may occur in this case.

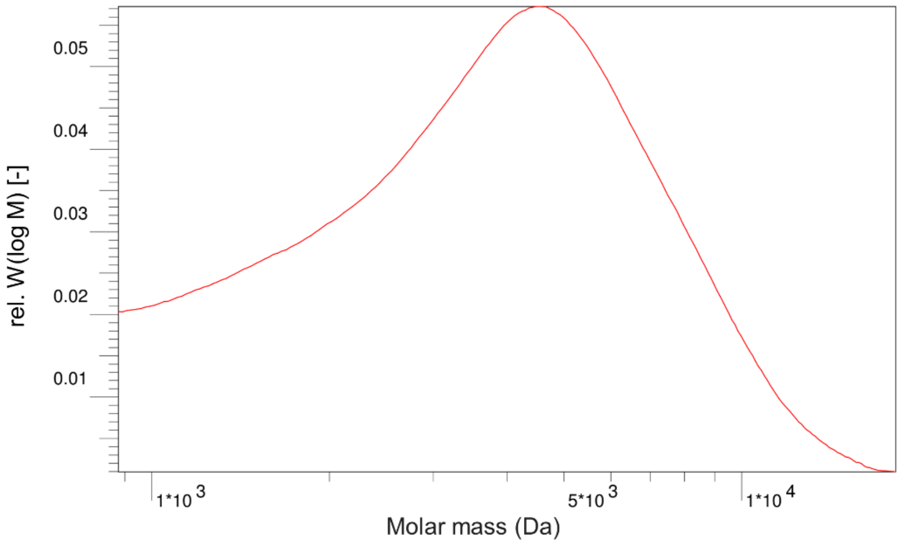

**$\overline{Mn}$:**	2.8169e3	g/mol	**$\overline{Mz}$:**	6.0019e3	g/mol
**Mw :**	4.2840e3	g/mol	**Mv :**	4.1089e3	g/mol
**D :**	1.5208e0				

Figure S27. Molecular weight distribution of Bu-CbzCys$_c$.

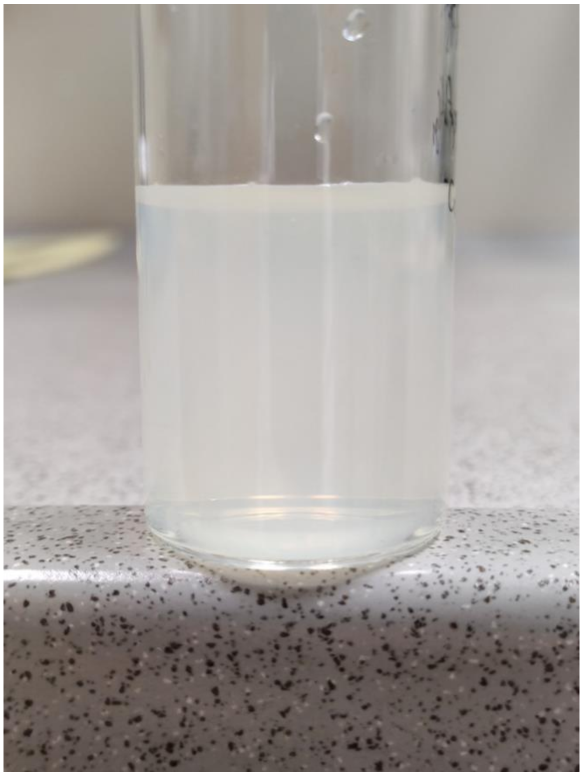

Figure S28. Photo of PFD filled peptide capsule dispersion.

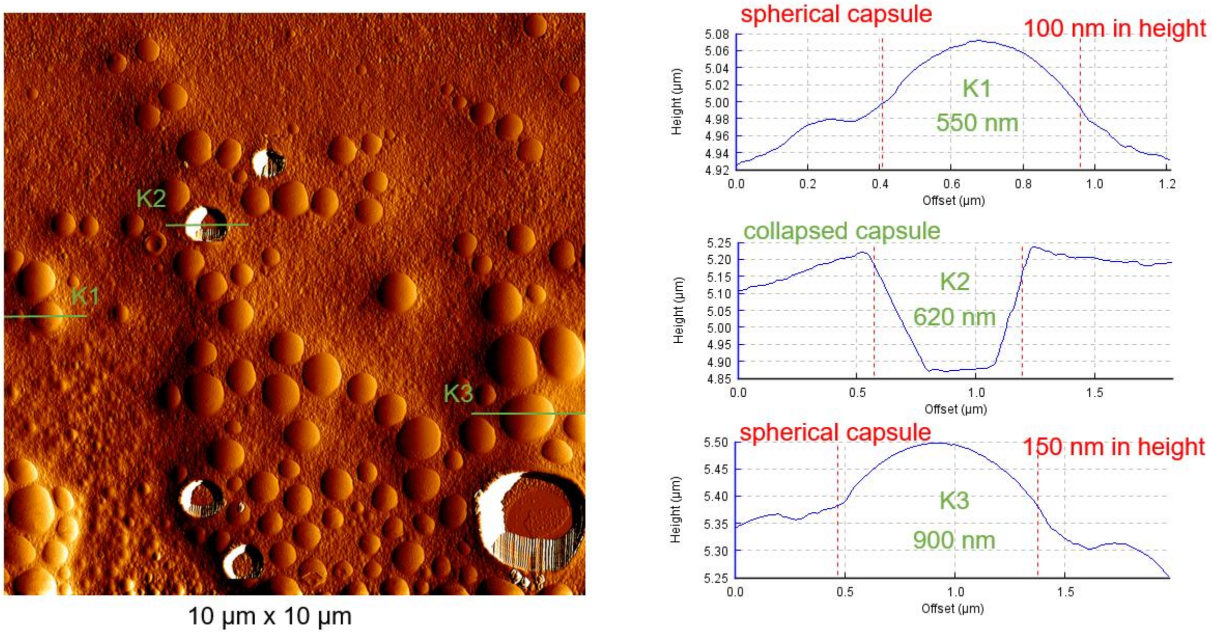

Figure S29. AFM image (left) and height profiles (right) of dried PFD-filled Bu-Asp_7-Cys_{15}-Phe_2 capsules from the dispersion sample stored for 3 weeks at room temperature.

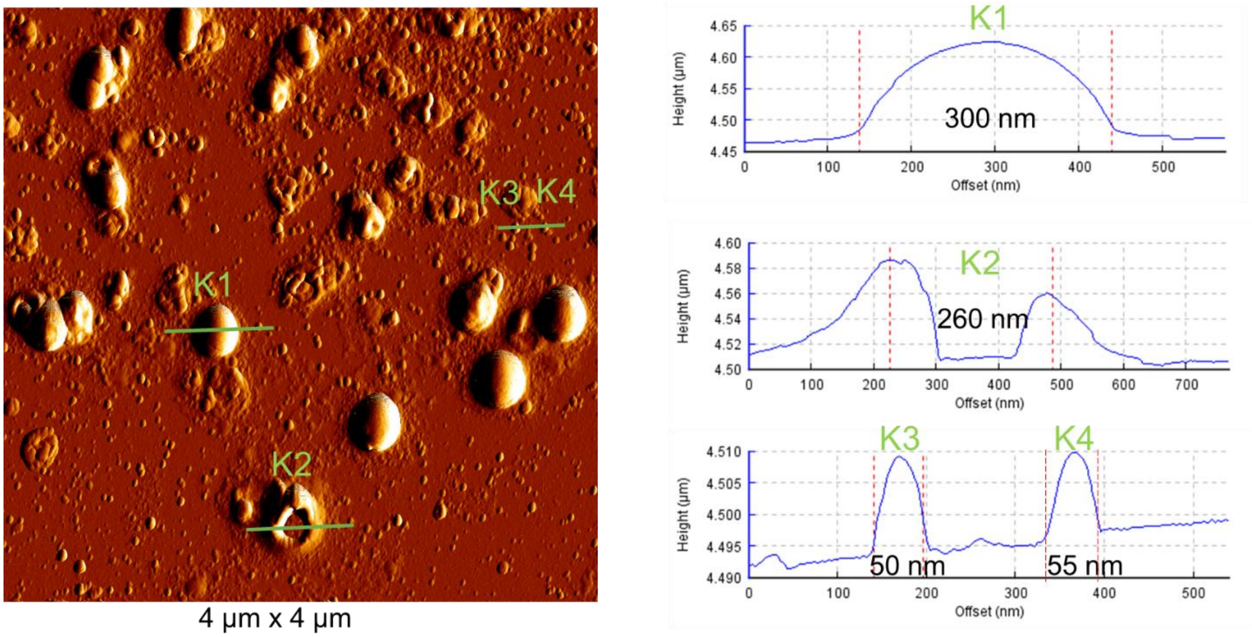

Figure S30. AFM image (left) and height profiles (right) of dried PFD filled Bu-Asp_6-Cys_8-Phe_8 capsules

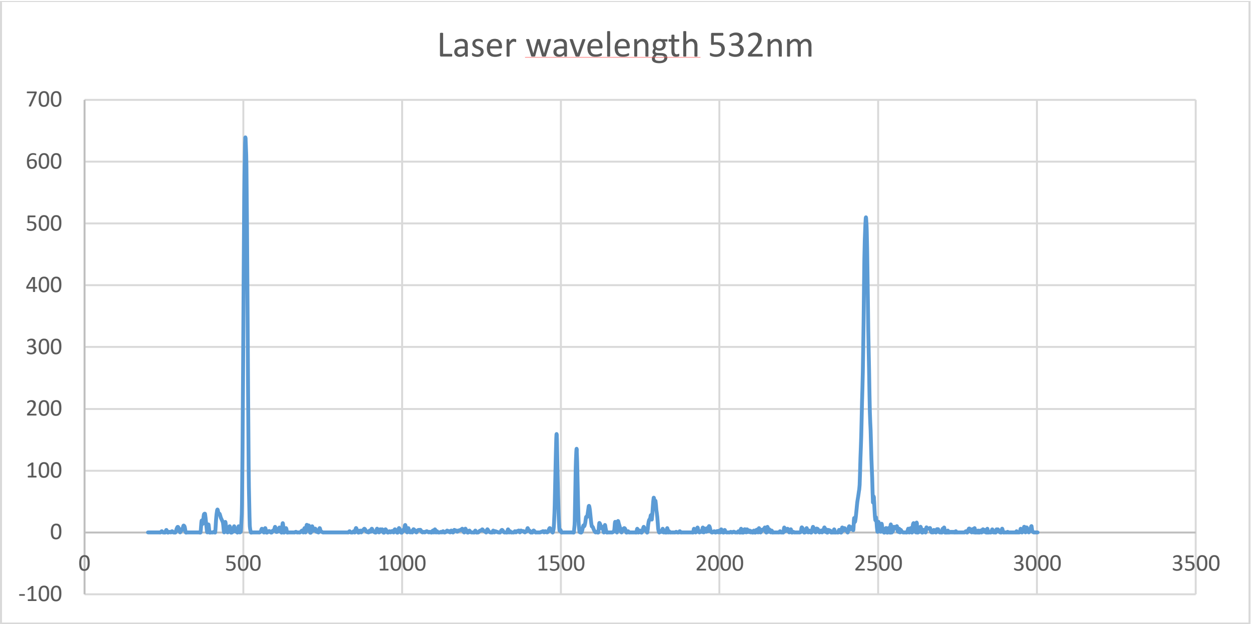

Figure S31. Raman spectrum of PFD-filled cysteine containing peptide capsules clearly showing the formation of disulfide bonds (506 nm).[1]

References

[1] J. Dai, S. D. Lin, D. Cheng, S. Y. Zou, X. T. Shuai, *Angew Chem Int Edit* **2011**, *50*, 9404-9408.
